# Flagellin peptide flg22 gains access to long-distance trafficking in Arabidopsis via its receptor, FLS2

**DOI:** 10.1093/jxb/erx060

**Published:** 2017-04-28

**Authors:** Joanna Jelenska, Sandra M. Davern, Robert F. Standaert, Saed Mirzadeh, Jean T. Greenberg

**Affiliations:** 1 Department of Molecular Genetics and Cell Biology, University of Chicago, Chicago, IL 60637, USA; 2 Biosciences Division, Oak Ridge National Laboratory, Oak Ridge, TN 37831, USA; 3 Nuclear Security and Isotope Technology Division, Oak Ridge National Laboratory, Oak Ridge, TN 37831, USA; 4 Department of Biochemistry & Cellular and Molecular Biology, University of Tennessee, Knoxville, TN 37996, USA; 5 Biology and Soft Matter Division, Oak Ridge National Laboratory, Oak Ridge, TN 37831, USA; 6 Shull Wollan Center — a Joint Institute for Neutron Sciences, Oak Ridge National Laboratory, Oak Ridge, TN 37831, USA

**Keywords:** Arabidopsis, flagellin, flg22, FLS2, long-distance traffic, peptide, *Pseudomonas syringae*, receptor-mediated endocytosis, transport.

## Abstract

Diverse pathogen-derived molecules, such as bacterial flagellin and its conserved peptide flg22, are recognized in plants via plasma membrane receptors and induce both local and systemic immune responses. The fate of such ligands was unknown: whether and by what mechanism(s) they enter plant cells and whether they are transported to distal tissues. We used biologically active fluorophore and radiolabeled peptides to establish that flg22 moves to distal organs with the closest vascular connections. Remarkably, entry into the plant cell via endocytosis together with the FLS2 receptor is needed for delivery to vascular tissue and long-distance transport of flg22. This contrasts with known routes of long distance transport of other non-cell-permeant molecules in plants, which require membrane-localized transporters for entry to vascular tissue. Thus, a plasma membrane receptor acts as a transporter to enable access of its ligand to distal trafficking routes.

## Highlight

Receptor-mediated endocytosis enables flg22 internalization, trafficking to vacuoles, entry to vasculature and mobilization to distal tissues. Receptors can function similarly to transporters and allow uptake and long-distance transport of molecules.

## Introduction

Metabolites and signaling molecules are transported to distal plant organs in vascular tissue (Lucas *et al.*, 2013). These molecules are translocated from cells where they are synthesized to the phloem via either symplastic (through plasmodesmata that connect cells) or apoplastic (extracellular) routes. In Arabidopsis, which is predominantly an apoplastic loader, assimilates such as sucrose are retrieved from the apoplast into phloem companion cell–sieve element complexes by transporters (Kühn and Grof, 2010; De Schepper *et al.*, 2013) and translocated from source to sink leaves with direct vascular connections. Such leaves occupy the same orthostichy, i.e. they are arranged in a vertical line on the same side of the stem (Kiefer and Slusarenko, 2003; Roberts *et al.*, 2007; Ferrieri *et al.*, 2015). Other molecules, including plant-derived peptides, viruses and defense signals, also move in phloem following vascular connections (Lough and Lucas, 2006). Systemic acquired resistance (SAR), induced by infection of lower leaves, is strongest in orthostichous leaves as well (Kiefer and Slusarenko, 2003; Lough and Lucas, 2006; Ferrieri *et al.*, 2015).

All secreted/apoplastic molecules that undergo long-distance transport have to enter phloem cells, usually via transporters. Viruses get inside the cells through damaged tissue or are transmitted by insect vectors directly to phloem cells (Vuorinen *et al.*, 2011; Hipper *et al.*, 2013). Whether other modes of entry, such as receptor-mediated endocytosis (RME), can provide access of ligands to phloem and long distance transport has not been addressed. We used a peptide pathogen associated molecular pattern (PAMP), flg22, to test this hypothesis. flg22 is a fragment of bacterial flagellin that binds the FLAGELLIN SENSITIVE 2 (FLS2) receptor (Gómez-Gómez and Boller, 2000) and induces interaction of FLS2 with its coreceptor, BRI1-ASSOCIATED KINASE 1 (BAK1) (Chinchilla *et al.*, 2007; Heese *et al.*, 2007; Sun *et al.*, 2013), and FLS2 endocytosis (Robatzek *et al.*, 2006). Subsequently, a consortium of defenses, including rapid generation of reactive oxygen species (ROS), is activated (Felix *et al.*, 1999; Zipfel *et al.*, 2004; Chinchilla *et al.*, 2006; Heese *et al.*, 2007). flg22 is widely used in basal defense studies. This 22-amino-acid peptide is buried within an assembled flagellum; it is not known how the flg22 epitope gets exposed to FLS2 and if during infection plants recognize intact flagellum, monomers or fragments of flagellin (Albert, 2013; Mott *et al.*, 2014).

Localization of FLS2 after activation by flg22 has been studied in epidermal cells in great detail (Robatzek *et al.*, 2006; Beck *et al.*, 2012; Choi *et al.*, 2013; Spallek *et al.*, 2013). Plasma membrane receptors such as BRASSIONOSTEROID INSENSITIVE 1 (BRI1) or, to a lesser extent, FLS2 are constantly endocytosed and are recycled back to the plasma membrane via the early endosome/trans-Golgi network (TGN) (Russinova *et al.*, 2004; Beck *et al.*, 2012). Ligand-activated receptors are sorted in the TGN and transported to late endosomes/multivesicular bodies (MVBs). After inducing a signaling cascade, activated FLS2 is ubiquitinated, endocytosed and degraded, transiently attenuating responsiveness to flg22 (Robatzek *et al.*, 2006; Lu *et al.*, 2011; Smith *et al.*, 2014). Degradation probably takes place within vacuoles, because ubiquitinated proteins removed from the plasma membrane by endocytosis are sorted in the TGN, and sent to MVBs and vacuoles for degradation (Scheuring *et al.*, 2012; Ben Khaled *et al.*, 2015). Castasterone (a brassinosteroid hormone) was shown to enter the endocytic pathway together with its receptor, BRI1, and to accumulate in vacuoles, while the receptor is recycled to the plasma membrane or degraded (Irani *et al.*, 2012; Martins *et al.*, 2015).

Recognition of flg22 by plants, in addition to causing an immediate local immune response that attenuates bacterial growth, can induce systemic resistance to subsequent infection (Mishina and Zeier, 2007). Application of other PAMPs such as lipopolysaccharide (LPS), chitosan or elicitins to the lower leaves of a plant also activates SAR (Ricci *et al.*, 1989; Mishina and Zeier, 2007; Faoro *et al.*, 2008; Zeidler *et al.*, 2010). It is assumed that secondary plant-derived mobile signals generated during the immune response to elicitors are responsible for SAR. However, PAMPs might also be transported to distal tissues. For example, *Salmonella minnesota* LPS infiltrated to Arabidopsis leaves was transported in vascular tissue (probably in xylem) to distal organs (Zeidler *et al.*, 2010) and *Phytophthora* elicitins were taken up by roots and transported to leaves in tobacco (Devergne *et al.*, 1992). Both types of PAMPs were internalized by plant cells: *Xanthomonas campestris* LPS was endocytosed and transported to vacuoles of *Nicotiana tabacum* suspension cells (Gross *et al.*, 2005) and elicitins were detected inside oak cells (Brummer *et al.*, 2002). So far, no connection between internalization and long-distance transport of PAMPs was made. Although flagellin uptake by plant cells has not been reported, endocytosis of externally applied flagellin was observed in human intestinal cells (Eaves-Pyles *et al.*, 2011).

Here we show that flg22 is transported to distal cells and tissues following vascular connections. Although flg22 is a synthetic peptide, we used it as a well-defined model of a peptide ligand that binds to the receptor and induces RME without endogenous background. We show that long-distance transport of flg22 depends on its receptor-mediated internalization into plant cells, raising the possibility that receptors facilitate delivery of mobile molecules to vascular tissue for distribution into distal organs.

## Materials and methods

### Labeled peptides

Unlabeled flg22 peptides and fluorescent derivatives labeled with 5-carboxytetramethylrhodamine (TAMRA) or 5-carboxyfluorescein (FAM) were ordered from Biomatik (95–98% purity) and are shown in Supplementary Table S1 at *JXB* online. Y-flg22 and Y-scrambled peptides were iodinated according to the method of McConahey and Dixon (1966). In brief, 20–75 µg of peptide was incubated with 2 mCi (17 Ci mg^–1^) of Na^125^I (Perkin Elmer) in the presence of chloramine T. The reaction was quenched by the addition of sodium metabisulfite, and 50 µl of BSA–phosphate-buffered saline (PBS) (5 mg ml^–1^) was added to the reaction vial prior to gel filtration chromatography. Labeled peptide was separated from free iodine by elution from a 5 ml Sephadex G-25 column pre-equilibrated in 5 mg ml^–1^ BSA–PBS. Fractions (~200 µl) were collected manually under gravity flow and counted for radioactivity. Peak fractions were pooled and labeling was confirmed by Tris-Tricine SDS-PAGE (10–20%). Wet gels, sealed in plastic and without prior staining, were exposed to autoradiography to retain any free iodine that might be present. Specific activity of labeling was 17–75 µCi µg^–1^.

### Plant material

*Arabidopsis thaliana* Col, *fls2* (Col) SALK_141277, *bak1-4* (Col) SALK_116202 and Ws and transgenic line FLS2-GFP (Ws) (Robatzek *et al.*, 2006) were grown at 20 °C with a 12 h light–12 h dark cycle for 4 weeks for most experiments, or on agar MS plates at 21 °C with a 16 h light–8 h dark cycle for 10–22 d for long-distance transport experiments with TAMRA–flg22. *Nicotiana benthamiana* grown for 5–6 weeks at 24 °C with a 16 h light–8 h dark cycle was used for peptide treatment 2 d after transient transformation with Agrobacterium GV3101 carrying FLS2–GFP (Robatzek *et al.*, 2006). Experiments with fluorescence and radioimaging were done in different laboratories (Chicago and Oak Ridge, respectively), with plants grown under the same conditions and treated at the same time of day.

### ROS assay

Bioactivity was verified for each batch of peptides by treating Col and *fls2* leaf discs (prefloated on water overnight) with 1 μM peptide and measuring ROS induction as described in (Tateda *et al.*, 2014).

### Subcellular localization of flg22

Leaf discs were floated (abaxial side down) on 1–5 μM TAMRA–flg22 or 5–10 μM FAM–flg22 solution (in water) at room temperature for the indicated time (lower concentrations were used for overnight treatments). Inhibitors that affect endocytosis, MG132 (Sigma-Aldrich, 50 μM) or 2,3-butanedione monoxime (BDM; Sigma-Aldrich, 50 mM) were added at the same time as the peptides. FM4-64 (Invitrogen, 6–8 μM) or 2′,7′-bis-(2-carboxyethyl)-5-(and-6)-carboxyfluorescein (BCECF)-AM (cleaved by an endogenous plant esterase to BCECF, Invitrogen, 20–40 μM) mixed with peptide solution was vacuum infiltrated for 2 min into leaf discs (Kang *et al.*, 2014) prior to incubation (floating on peptide/dye solution) for the indicated time. In some experiments FAM–Y-flg22 was used with results similar to FAM–flg22.

For experiments with mixed peptides, leaf discs were floated for the indicated time on 2 μM FAM–flg22, FAM–scrambled peptide or fluorescein mixed with 1 μM TAMRA–flg22. In all experiments, leaf discs were washed after treatment by gently shaking for several minutes in water and the abaxial side was imaged by confocal or epifluorescence microscopy (see ‘Microscopy’). Fluorescence intensity profiles were generated with ImageJ, as in Spallek *et al.*, (2013) and Bozkurt *et al.* (2015) (see ‘Data analysis’). Co-localization of flg22 and FLS2 was observed in three experiments and co-localization of flg22 and dyes in two experiments, with at least three plants per genotype/treatment/time point.

### Microscopy

Epifluorescence images were obtained with a Leica DMR microscope (Leica, Germany) using green fluorescent protein (GFP) and rhodamine filter sets.

Confocal images were obtained using a Zeiss LSM710 laser-scanning confocal microscope (Zeiss, Germany) as described in Kang *et al.* (2014). Fluorescence was visualized as follows: GFP/FAM/fluorescein/BCECF excitation (ex) 488 nm/emission (em) 495–545 nm; TAMRA ex 561 nm/em 566–620 nm; FM4-64: ex 561 nm/em 686–758 nm. Green and red fluorescence was acquired for the same field using a sequential acquisition mode.

In each experiment, all images were obtained with the same settings to allow comparison between samples. Imaging was done at the same time of the day for all experiments. Representative images are shown. Multiple images were used for quantification and were analysed as described below (see ‘Data analysis’).

### Long-distance transport of flg22

#### Movement across a leaf blade 

A 5 μl drop of water solution of 10 μM FAM–flg22 or FAM–scrambled peptide was placed on the abaxial side of a detached Arabidopsis leaf in a humid chamber. In some experiments FAM–Y-peptides were used with similar results. Excess peptide was removed after 0.5 h, leaf surface was washed with water and epifluorescence images of the adaxial side were taken during the next 30 min (0.5–1 h after treatment).

#### Long-distance transport to distal tissues

##### Infiltration 

A 5–10 μM water solution of FAM–flg22 was pressure infiltrated with a 1 ml blunt syringe into a part or all of one leaf of 4-week-old soil-grown plants and distal tissues were examined by epifluorescence and confocal microscopy after 2 and 16 h, respectively. FAM–Y-flg22 was used in some experiments with similar results.

##### Surface application of fluorescent peptide 

We applied 10–20 μl of 5 μM TAMRA–flg22 (without or with inhibitors: 50 μM MG132 or 50 mM BDM) to a filter paper disc placed on the adaxial side of one leaf of *in vitro*-grown Arabidopsis plants and distal tissues were imaged by confocal microscopy after 1 d. For experiments with mixed peptides, 5 μM TAMRA–flg22 + 10 μM FAM–scrambled peptides were used.

##### Surface application of radiolabeled peptide 

We applied 0.5–1 µM (specific activity 17 µCi µg^–1^) ^125^I-Y-flg22 or ^125^I-Y-scrambled peptide in 10 mM MES pH 5.7 with 0.01% Tween-20 as a 1 µl droplet to the abaxial side of one mature leaf of 4-week-old soil-grown Arabidopsis plants and incubated for 3 h. For the control, 1 µl of 1.8 µM Na^125^I (2.2 µCi µg^–1^) in 5 mM NaOH–0.5 mM NaHSO_2_ (to prevent volatilization) with 0.005% Tween-20 was applied in the same way. Whole plants were sealed in plastic (Pinnacle Cover-All, Total Care), additionally contained in a plastic bag, and exposed for 5 d to phosphor screens for autoradiography.

In each experiment, all images were obtained with the same settings to allow comparison between samples. Fluorescence and radioactivity were quantified as described below (see ‘Data analysis’). flg22 movement was examined in at least three experiments of each type; see figure legends for details.

### Analysis of the stability of radiolabeled peptides in plants and extracts

Radiolabeled peptides (in 10 mM MES pH 5.7 with 0.01% Tween-20) were applied (i) as a 5 µl droplet (3 µM peptide, 75 µCi µg^–1^) to the abaxial surface on one leaf on an intact plant, (ii) into the whole plant through a tube attached to the cut petiole of a fully expanded Col leaf (5.7 µM peptide, 75 µCi µg^–1^), or (iii) into individual leaves through a cut petiole (3 µM peptide, 17 µCi µg^–1^). For control, 1 μl droplet of 0.2–1.8 µM Na^125^I (1.1–2.2 µCi µg^–1^) in 5 mM NaOH–0.5 mM NaHSO_2_ and 0.005% Tween-20 was applied to the abaxial surface on one leaf on an intact plant. After 2–3 h, local and distal leaf tissues were homogenized in 50–100 µl of 2% SDS–PBS.

Alternatively, radiolabeled peptides (2–10 µM, 17–75 µCi µg^–1^) or Na^125^I (0.2 µM, 1.1 µCi µg^–1^) were incubated for 2.5–3 h with fresh or boiled (for 10 min) leaf extracts in PBS with 0.2% Igepal CA-630. Peptides were analysed by Tris-Tricine SDS-PAGE (10–20% gel, Bio-Rad) and visualized by autoradiography (20 min to 20 h exposure).

These experiments were repeated at least twice, with duplicates or triplicates.

### Sucrose uptake and transport

Arabidopsis leaf discs were floated on 5 μCi ml^–1^ (435 mCi mmol^–1^) [^14^C]sucrose in 1 mM MES pH 5.6 with or without 2 μM unlabeled peptide for 1.5 h, washed four times with buffer during 1 h, dried, and radioactivity was measured in a scintillation counter (LS 6000 IC, Beckman) (Zhang and Turgeon, 2009).

For phloem flow evaluation, a 20 μl drop containing 0.2 μCi [^14^C]sucrose in 1 mM MES pH 5.6 with or without 10 μM flg22 was applied on the abaxial side of one leaf of soil-grown plants and after 16 h rosettes were removed, flattened on a filter paper and imaged by autoradiography (6 d exposure at –70 °C). Later, radioactivity in individual leaves was quantified by scintillation counting.

These experiments were repeated at least twice.

### Data analysis

Fluorescence intensity profiles were generated with ImageJ (http://rsb.info.nih.gov/ij, last accessed 6 April 2017) using RGB Profiler and Color Profiler. Fluorescent puncta were manually counted in 20 μm × 20 μm confocal image areas as in Irani *et al.* (2012) and Chaparro-Garcia *et al.*, (2015) and shown as a fraction (Irani *et al.*, 2012; Choi *et al.*, 2013).

The amount of peptide in plant cells was measured as total fluorescence per view field (integrated density) as in Martins *et al.* (2015), and was quantified with ImageJ in each channel in confocal and epifluorescence images. To allow comparison between experiments, fluorescence was calculated as a percentage of the signal in Col treated with TAMRA–flg22 or FAM–flg22. ^125^I radioactivity in local and distal tissues was quantified from autoradiographs using a Cyclone Phosphorimager (Perkin Elmer) or in a scintillation counter (Packard Quantum Cobra D5002) and shown as percentage of total radioactivity in a plant. [^14^C]sucrose radioactivity was quantified in a scintillation counter. Data from multiple experiments were analysed together using Prism software (GraphPad) (one-way ANOVA followed by Tukey’s test, or the *t*-test).

## Results

### Labeled flg22 is biologically active

To study the fate of flg22 at the whole plant level, we used both fluorescent and radiolabeled peptides. flg22 and a control peptide with a scrambled flg22 sequence were labeled at the N-termini with 5-carboxyfluorescein (FAM), 5-carboxytetramethylrhodamine (TAMRA) or ^125^I to track peptide movement in plant tissues (see Supplementary Table S1). A tyrosine residue, required for iodination, was added at the N-termini of some peptides (Meindl *et al.*, 2000). All of the labeled flg22 peptides retained biological activity and induced ROS in an FLS2-dependent manner, whereas all of the scrambled flg22 derivatives were inactive (see Supplementary Fig. S1).

### flg22 is mobile in plants and its long-distance movement requires FLS2–BAK1 coreceptors

**Fig. 1.**
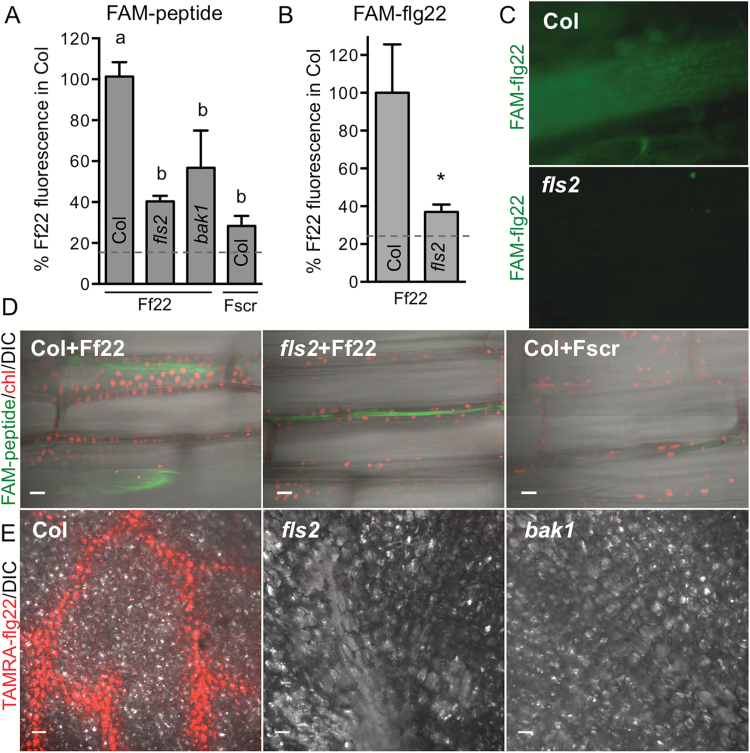
flg22 transport in plants requires FLS2 receptor. flg22 is detected in vascular regions. (A) FAM–flg22 (Ff22) was detected on the adaxial side of a detached Col leaf 1 h after drop application of 10 μM peptide to the abaxial side. Migration of FAM–scrambled peptide (Fscr) in Col and FAM–flg22 in *fls2* and *bak1* was significantly lower. FAM fluorescence was quantified in epifluorescence microscopy images of adaxial (distal) leaf side; data combined from three experiments are shown as percentage of FAM–flg22 signal in Col. Background fluorescence (mock treatment without peptides) is shown by a dashed line. Letters indicate significant difference (*P*<0.01, ANOVA/Tukey’s test, *n*≥5). (B, C) FAM–flg22 transport to distal leaves is less efficient in the *fls2* mutant. FAM–flg22 or FAM–Y-flg22 (5–10 μM) was infiltrated into one leaf of soil-grown plants, and fluorescence was quantified after 16 h in epifluorescence microscopy images of distal orthostichous leaves (such as in C); data combined from three experiments are shown as percentage of signal in Col. *P<0.02 (*t*-test, *n*=12). Background fluorescence is shown by dashed line. (D) Two hours after infiltration of 10 μM FAM–flg22 or FAM–Y-flg22 into a part of Col leaf, FAM fluorescence was detected inside vascular cells outside the infiltrated area. FAM–flg22 in *fls2* and FAM–scrambled peptide in Col were rarely detectable outside infiltration area and only between the cells. A composite of confocal microscopy image with differential interference contrast (DIC), green FAM fluorescence and red chloroplast (chl) autofluorescence is shown. Bar: 20 μm. (E) TAMRA–flg22 is detected predominantly in vascular region of Col. Five micomolar TAMRA–flg22 was applied on a filter paper disc placed on the adaxial side of a leaf of *in vitro*-grown Arabidopsis plants and confocal images of treated tissue (adaxial side underneath filter paper) were obtained 1 d later, after washing the leaf with water. The distinct vein pattern in Col was observed in 4 out of 7 experiments. Bar: 50 μm.

We first assessed whether flg22 shows short- and/or long-distance movement in plants using several methods of peptide delivery. FAM–flg22 was detected on the adaxial side of a detached Col leaf 1 h after droplet application to the abaxial side, indicating movement over a short range within the same organ (Fig. 1A). FAM–flg22 mobility across a leaf blade was reduced in *fls2* and *bak1* mutants and was similar to the mobility of the FAM-labeled scrambled peptide in Col (Fig. 1A). Sixteen hours after FAM–flg22 or FAM–Y-flg22 was infiltrated into a part of a leaf, the peptide was detected in distal orthostichous Col leaves, especially in veins. In contrast, FAM fluorescence was not observed in distal *fls2* leaves (Fig. 1B, C). At a shorter time, 2 h after infiltration, no fluorescence could be detected in distal organs, but FAM–flg22 was present within vascular cells in a distal part of the infiltrated Col leaf. FAM–flg22 in *fls2* and FAM–scrambled peptide in Col were not detected within cells. Fluorescence signals were only rarely detected in intercellular spaces in distal areas of infiltrated leaves (Fig. 1D).

**Fig. 2.**
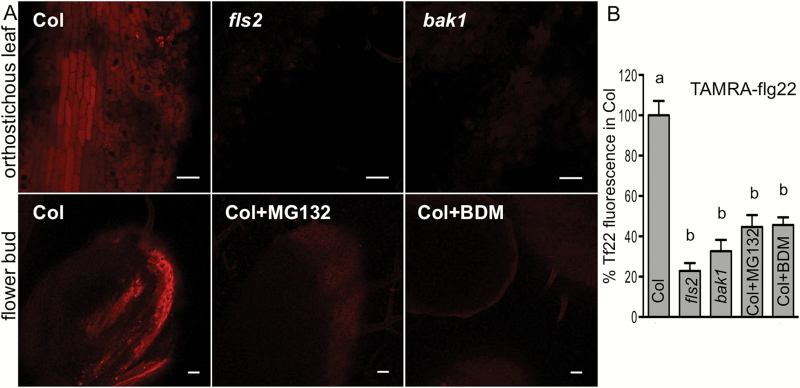
flg22 long-distance movement depends on receptors and internalization. Five micromolar TAMRA–flg22 (Tf22) with or without inhibitors was applied on a filter paper disc placed on one leaf of *in vitro*-grown Arabidopsis, and orthostichous distal tissues (leaves and flowers) were imaged by confocal microscopy after 1 d. TAMRA–flg22 was transported more efficiently to distal tissue in Col than in *fls2* and *bak1* or in Col treated with inhibitors. (A) Representative confocal microscopy images of distal tissues 1 d after peptide application. Bar: 50 μm. (B) TAMRA fluorescence was quantified in images such as in (A) from at least four experiments (except BDM treatment in two experiments) and presented as percentage of fluorescence in distal tissue of Col. Letters indicate significant difference (*P*<0.0002, *n*≥7, ANOVA/Tukey’s test).

During bacterial infection, flagellin is likely to be first detected by the plant on leaf or stem surfaces. FLS2 is active in the epidermis, as flg22 was shown to induce calcium signals and oscillations in leaf epidermal cells (Thor and Peiter, 2014; Keinath *et al.*, 2015). Therefore, in subsequent experiments we delivered flg22 to a leaf surface. Droplet application of FAM- or TAMRA-labeled peptides to one leaf of large soil-grown plants did not allow detection of fluorescence signal in distal leaves. Therefore, we applied TAMRA-peptides on a Whatman paper disc placed on the adaxial side of one leaf of *in vitro*-grown plants, which allowed larger contact area and prolonged delivery of a peptide to much smaller plants. One day after application TAMRA–flg22 was detected preferentially in the vascular region of Col local tissue, but not in *fls2* or *bak1* (Fig. 1E). TAMRA–flg22 was also detected after 1 d in distal leaves and flowers of Col (Fig. 2). The efficient transport of flg22 required the receptors, as significantly less flg22 was detected in distal organs of *fls2* and *bak1* mutant plants (Fig. 2A, B). The differences in peptide accumulation in distal tissues observed in the microscopy images were statistically significant (Figs 1A, B and 2B). Thus, efficient long-distance transport of flg22 peptide is enabled by the FLS2 receptor. Additionally, several different methods (drop application, infiltration and contact with peptide-soaked filter paper) yielded similar results.

### Long distance movement of ^125^I-Y-flg22 follows plant orthostichy

**Fig. 3.**
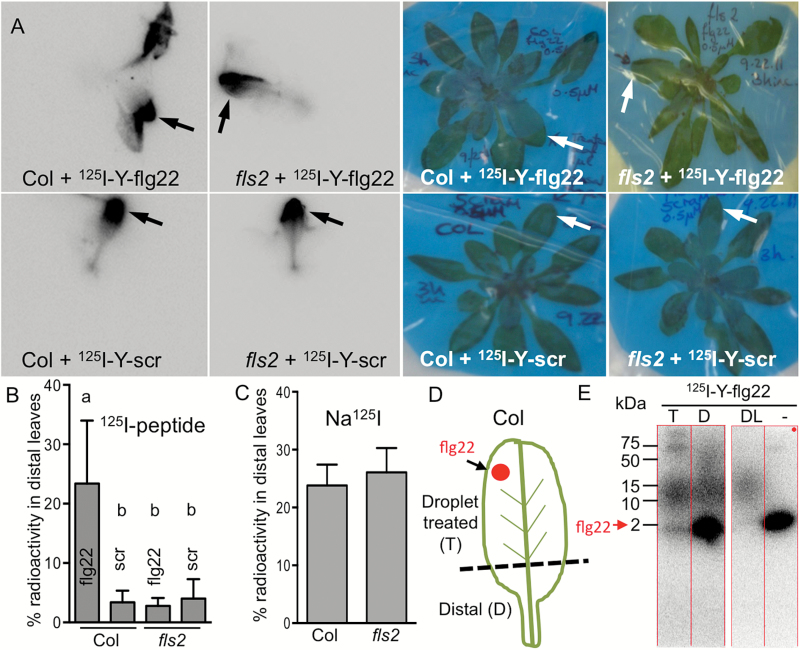
flg22 movement is specific and FLS2-dependent. Labeled peptide is not degraded in plants. (A, B) ^125^I-Y-flg22 is detected 3 h after application in distal orthostichous leaves of Col but not *fls2*. Scrambled peptide (scr) is not mobile. A 1 μl droplet of 0.5 μM radiolabeled peptide was applied to the abaxial side of one leaf (arrow) of plants grown in soil and imaged by autoradiography after 3 h (A). The highest signal was detected in orthostichous leaves of flg22-treated Col. Percentage of radioactivity in distal leaves was quantified in six plants from four experiments (B). Letters indicate significant difference (*P*<0.05, ANOVA). (C) Transport of control Na^125^I is not affected by FLS2. A 1 μl droplet of Na^125^I (1 × 10^6^ cpm) was applied to the abaxial side of one leaf (as in A) and plants were incubated for 3 h. ^125^I signal was quantified in distal leaves of five plants from three experiments (*P*=0.7, *t*-test). (D, E) flg22 is not degraded and is detected in distal parts of droplet-treated Col. Five microliters of 3 μM ^125^I-Y-flg22 was applied to Col plants (as in A) and after 3 h the treated leaf was removed and cut in two parts (dashed line, D). Proteins extracted from both parts of the treated leaf and from distal orthostichous leaves were separated by SDS-PAGE and detected by autoradiography (E). Parts of the same gel (same exposure) are shown in (E). T, extract from treated leaf segment (0.13% of sample); D, extract from distal region of the treated leaf (40% of sample); DL, extract from distal orthostichous leaves (80% of sample); –, control ^125^I-Y-flg22 (not extracted from plants). Unmodified ^125^I-Y-flg22 (~2 kDa, red arrow) and larger band (~12 kDa) were detected in extracts from treated and distal tissue of five plants in two experiments.

Experiments using ^125^I-labeled peptide confirmed the mobility of flg22 in Arabidopsis. Higher sensitivity and lower background allowed detection of the radiolabeled peptide in distal tissues of soil-grown Arabidopsis 3 h after droplet application to the abaxial side of one leaf (Fig. 3A, B). This method also allowed us to image whole plants and to determine the actual distribution of a peptide in a plant. On average, 23% of total ^125^I-Y-flg22 moved to distal leaves of Col. We observed orthostichous movement of ^125^I-Y-flg22 to leaves on the same side of the plant (Kiefer and Slusarenko, 2003; Roberts *et al.*, 2007) as the treated leaf (Fig. 3A and Supplementary Figs S2 and S3). This pattern of movement suggests that flg22 is transported by the vascular tissue and it is consistent with preferential detection of fluorescent flg22 in a vascular tissue (Fig. 1C–E).

As seen with fluorophore-labeled peptide, long-distance movement of ^125^I-Y-flg22 was dependent on FLS2 (Fig. 3A, B, and Supplementary Figs S2 and S3). Furthermore, the mobility of the scrambled peptide was reduced in both Col and *fls2* plants to a similar extent as flg22 in *fls2* (Fig. 3A, B). Na^125^I movement, used as a control, was the same in Col and *fls2* (Fig. 3C).

We analysed peptides in extracts from treated and distal plant tissues by SDS-PAGE to test their stability. While signals from fluorescent peptides were below the detection limit, unmodified ^125^I-Y-flg22 and a larger 12 kDa complex were detected in the extracts (Fig. 3D, E and Supplementary Fig. S4A, B). In earlier work with tomato cell suspension culture, ^125^I-Y-flg22 associated with the cells was not degraded either (Meindl *et al.*, 2000). We did not observe release of radiolabel from ^125^I-Y-flg22 in Arabidopsis plant tissue. The larger (12 kDa) band was not sequence or receptor dependent (see Supplementary Fig. S4C). It was also observed after peptide incubation with plant protein extracts, but not with heat-treated extracts (Supplementary Fig. S4D), and thus possibly it was formed during extract preparation, due to the mixing of the contents of subcellular compartments. Free Na^125^I did not form any complexes in plants or in plant extracts; therefore, it is unlikely that Na^125^I became detached from the peptide and bound to another molecule in the plant extract (Supplementary Fig. S4E, F).

Our data indicate that radiolabeled peptide is not degraded in distal tissues and at least some flg22 is mobile in the unmodified form. Experiments with radiolabeled peptides further support a specific role of the receptors in orthostichous transport of flg22. Importantly, experiments with fluorescent and radiolabeled peptides were done independently and in parallel in two different locations without prior knowledge of experimental outcomes from either location. Both sets of experiments were in agreement that flg22 requires its receptor for long distance transport and scrambled peptides do not show long distance movement. In all experiments that used different peptide application and detection techniques, unspecific movement (in *fls2* or with scrambled peptide) was ~15–20% of FLS2-dependent movement of active flg22 peptide (Figs 1B, 2B, 3B and 8B).

### flg22 is internalized together with FLS2 by plant cells

Extracellular molecules must first be internalized into cells in order to be transported in vascular tissue to distal organs. Since FLS2 is needed for the long distance movement of flg22, we hypothesized that flg22’s movement should be correlated with the ability of the peptide to enter cells. Therefore, we tested if FLS2 enables internalization (uptake) of flg22.

**Fig. 4.**
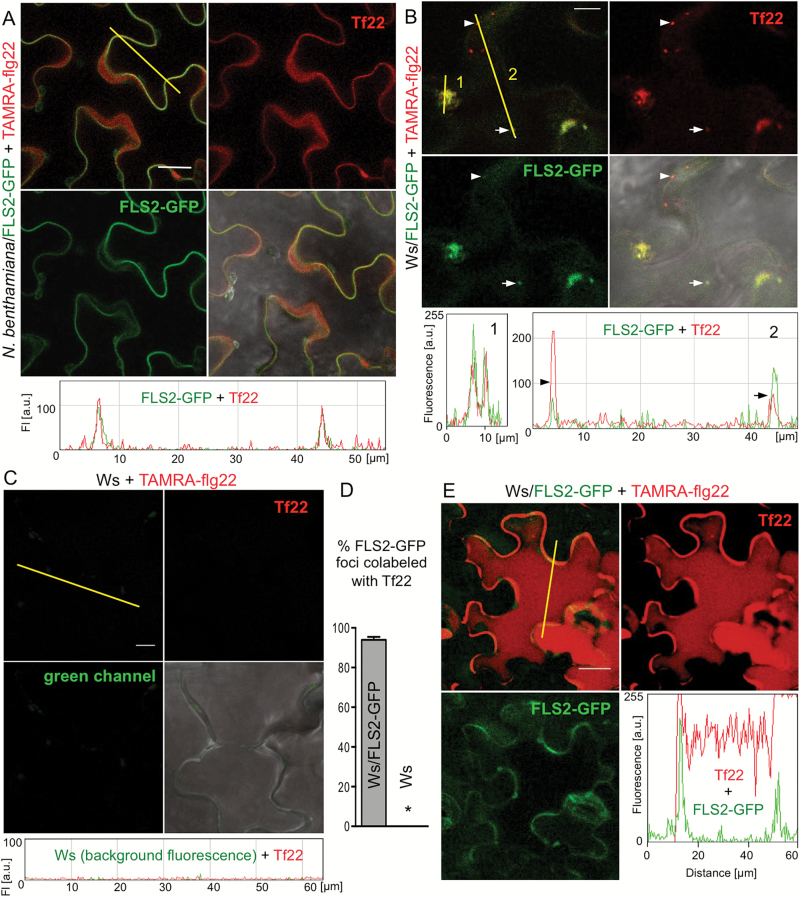
flg22 is internalized together with FLS2. Leaf discs of *N. benthamiana* (A) and Arabidopsis Ws expressing FLS2–GFP (B, E) or control Ws (C) were floated on 2 μM TAMRA–flg22 (Tf22) for the indicated time, washed in water and imaged by confocal microscopy. Images in red (TAMRA) and green (GFP) channels, and their composite with or without DIC image are shown. Graphs show fluorescence profiles along yellow line. Bar: 20 μm in (A, E) and 10 μm in (B, C). These experiments were repeated three to four times. (A) TAMRA–flg22 colocalizes at plasma membrane with FLS2–GFP after several minutes to 1 h incubation. See additional images in Supplementary Figs S5 and S6. (B) TAMRA–flg22 is internalized and colocalizes with FLS2–GFP in vesicles (line 2, two vesicles along the line marked with an arrowhead and an arrow) and MVBs (line 1) after 1–2 h incubation. See additional images in Supplementary Fig. S7. (C) TAMRA–flg22 does not enter wild-type Ws cells that lack FLS2 after 1–2 h incubation. See additional images in Supplementary Figs S6 and S7. (D) TAMRA–flg22 colocalizes with FLS2–GFP after floating Ws/FLS2–GFP leaf discs on peptide solution for 1–2 h. Fluorescent foci were counted in 16 picture areas (20 μm × 20 μm) for each genotype. Total number of foci: Ws/FLS2–GFP: 152 green (FLS2–GFP), 161 red (Tf22), 143 overlap; Ws: 2 green, 5 red, 0 overlap. Error bars, SE, *P<0.0001 (*t*-test, *n*=16). (E) After overnight incubation, TAMRA–flg22 fills the cells, whereas FLS2–GFP is mostly detected at the plasma membrane.

We used confocal microscopy to examine the localization and fate of flg22 in plant cells at the site of application. Leaf discs of a FLS2–GFP line of Arabidopsis Ws, which lacks endogenous functional FLS2 (Robatzek *et al.*, 2006), and *Nicotiana benthamiana* transiently transformed with FLS2–GFP were floated on TAMRA–flg22, washed in water and imaged. TAMRA–flg22 and FLS2–GFP co-localization at the plasma membrane was observed in *N. benthamiana* after several minutes to 1 h incubation (Fig. 4A and Supplementary Fig. S5) and in Arabidopsis Ws after 15–20 min (Supplementary Fig. S6). After 1–2 h, TAMRA–flg22 and FLS2–GFP were found together in vesicles in Ws/FLS2–GFP (Fig. 4B, D and Supplementary Fig. S7). FLS2–GFP was also internalized and found in vesicles in Ws/FLS2–GFP after 1–1.5 h treatment with unlabeled flg22 and TAMRA–flg22 was found in vesicles in Col (which has a wild-type, unlabeled FLS2) (see Supplementary Fig. S7). No fluorescence signal was observed inside the cells of the non-transgenic wild-type Ws control or Col *fls2* treated with TAMRA–flg22 (Fig. 4C, D and Supplementary Fig. S7) or in wild-type Col or Ws treated with unlabeled flg22 (see Supplementary Fig. S7).

After 16 h incubation, TAMRA–flg22 co-localized with FLS2–GFP at the plasma membrane. Fluorescence signals from TAMRA, but not GFP, also appeared to fill the cells, suggesting flg22 accumulation in large central vacuoles, which occupy most of the volume of Arabidopsis epidermal cells (Fig. 4E) (Hunter *et al.*, 2007). The lack of GFP signal in vacuoles probably reflects the fact that FLS2 is degraded after internalization in response to flg22 (Tamura *et al.*, 2003; Robatzek *et al.*, 2006; Lu *et al.*, 2011; Smith *et al.*, 2014).

In all experiments we imaged flg22 in epidermal cells, similarly to how it was done for FLS2 (Robatzek *et al.*, 2006; Beck *et al.*, 2012; Choi *et al.*, 2013; Spallek *et al.*, 2013), but flg22 accumulated in mesophyll cells as well (see Supplementary Fig. S8). Our experiments show that flg22 is internalized with its receptor FLS2.

### flg22 traffics in the endocytic pathway to vacuoles

**Fig. 5.**
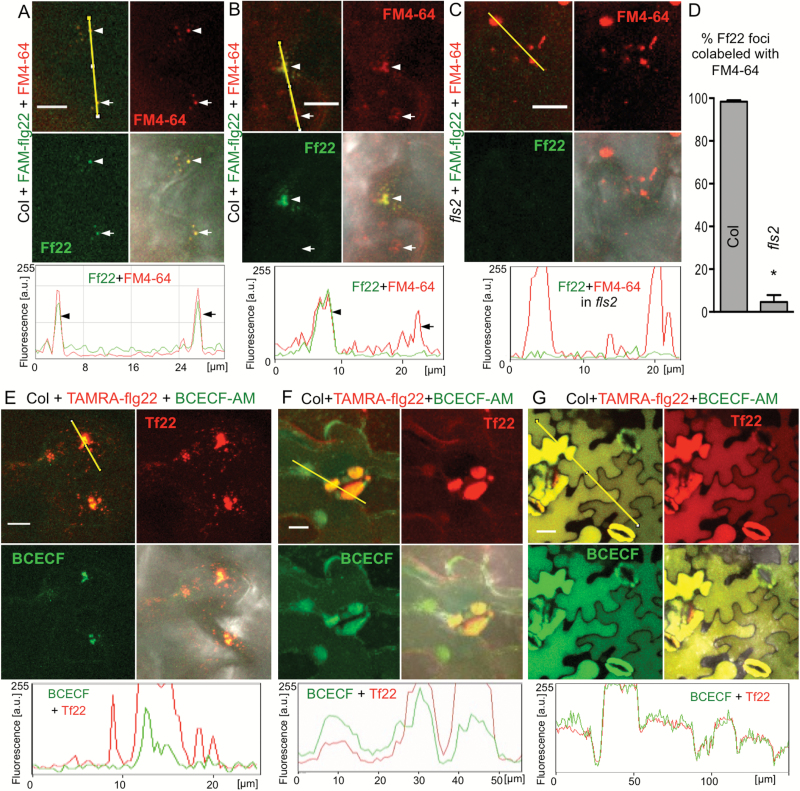
flg22 undergoes endocytosis and accumulates in vacuoles. Col leaf discs were vacuum infiltrated with fluorophore-labeled flg22 and a dye prior to floating on flg22/dye solution for the indicated time. Confocal microscopy images in red (TAMRA, FM4-64) and green (FAM, BCECF) channels and a composite of fluorescence channels with or without DIC are shown. Graphs show fluorescence profiles along yellow line. Bar: 10 μm in (A–C, E, F) and 20 μm in (G). These experiments were repeated twice. (A) FAM–flg22 (Ff22, 5–10 μM) colocalizes in vesicles with plasma membrane/endosome dye FM4-64 (6–8 μM) after 1 h incubation. (B) Later (after 1–2 h), FAM–flg22 colocalizes with FM4-64 in endosomes and MVBs (arrowhead). Not all endosomes and MVBs contain FAM–flg22 (arrow). (C) FAM–flg22 is not detected inside *fls2* cells after 1–2 h incubation. (D) FAM–flg22 colocalizes with endocytic dye FM4-64 in Col leaf discs after 1–2 h incubation. Fluorescent foci were counted in 18 picture areas (20 μm × 20 μm) for each genotype. Total number of foci: Col: 217 green (Ff22), 325 red (FM4-64), 212 overlap; *fls2*: 10 green, 289 red, 2 overlap. Error bars, SE, *P<0.0001 (*t*-test, *n*=18). (E, F) TAMRA–flg22 (Tf22, 5 μM) colocalizes with the vacuolar dye BCECF (40 μM) in MVBs (E) and prevacuolar compartments (F) after 2–4 h incubation. TAMRA–flg22 is also found in endosomes (narrow red peaks in fluorescence profile) moving to MVBs (E). (G) After overnight incubation, TAMRA–flg22 (1 μM) colocalizes with BCECF (20 μM) in central vacuoles.

Cellular trafficking of flg22 and the identity of fluorescently labeled compartments was evaluated by colocalization with dyes staining plasma membrane, endocytic vesicles and MVBs (FM4-64) (Bolte *et al.*, 2004; van Gisbergen *et al.*, 2008) or prevacuolar compartments/vacuoles (BCECF) (Brauer *et al.*, 1995; Scheuring *et al.*, 2015). FM4-64 has previously been used to confirm endocytosis of activated FLS2 (Beck *et al.*, 2012). FAM–flg22 co-localized with a subset of vesicles and MVBs labeled with the membrane/endocytic dye FM4-64 in Col leaves after 1–2 h incubation (Fig. 5A, B, D). As expected, FAM–flg22 signal was not visible in the *fls2* mutant (Fig. 5C, D). After 2–4 h, TAMRA–flg22 was found in MVBs and prevacuolar compartments stained with the vacuolar dye BCECF (endosomes were not stained with BCECF) (Fig. 5E, F). After overnight incubation, TAMRA–flg22 and vacuolar dye accumulated in central vacuoles, filling a large volume of the cells (Fig. 5G).

In conclusion, flg22 follows the default endocytic pathway to vacuoles typical for plasma membrane proteins (Scheuring *et al.*, 2012) and similar to brassinosteroids (Irani *et al.*, 2012).

### flg22 accumulation in plant cells requires endocytosis and FLS2–BAK1 coreceptors

**Fig. 6.**
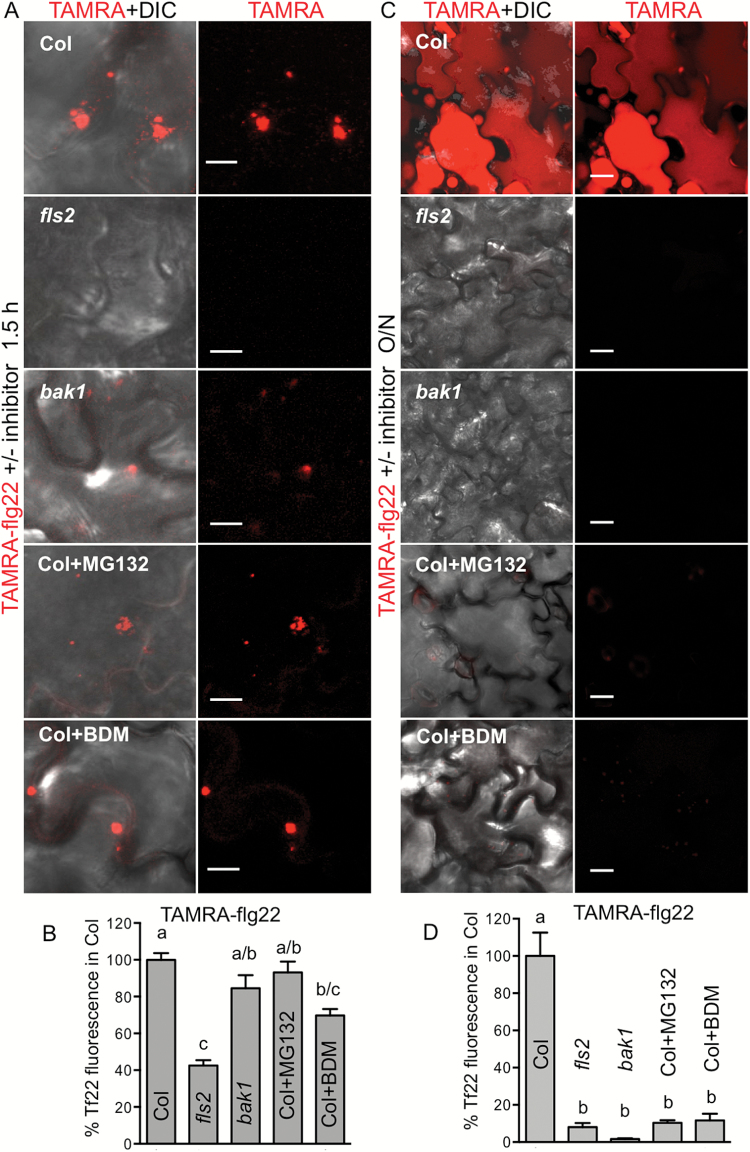
Accumulation of flg22 in plant cells requires FLS2 and endocytosis. Arabidopsis leaf discs were floated on TAMRA–flg22 (Tf22) with and without an endocytosis inhibitor for 1–2 h (A, B; 5 μM TAMRA–flg22) or overnight (C, D; 1–2 μM TAMRA–flg22) and washed in water. Fifty micromolar MG132 or 50 mM BDM was added simultaneously with TAMRA–flg22. Fluorescence was quantified in confocal microscopy images such as in (A, C); data were combined from at least two experiments for each genotype/treatment and shown as percentage of TAMRA–flg22 signal in Col. Letters indicate significant difference (ANOVA/Tukey’s test). Background fluorescence in the absence of TAMRA–flg22 was negligible. Bar: 10 μm in (A) and 20 μm in (C). (A, B) Initial (up to 1.5 h) uptake/binding of TAMRA–flg22 is reduced in *fls2*, but not highly affected in *bak1* or in Col treated with inhibitors. *P*<0.05, *n*≥6. (C, D) Overnight accumulation of TAMRA–flg22 in plant cells is inhibited in *fls2* and *bak1* and in Col treated with inhibitors. *P*<0.0002, *n*≥7.

Since we did not detect internalization and accumulation of flg22 in cells of Ws (which lack FLS2) and an *fls2* mutant of Col (Figs 4C, D and 5C, D, and Supplementary Fig. S7), we hypothesized that flg22 enters plant cells via RME. Therefore, we further investigated factors required for flg22 uptake and accumulation. We quantified binding/initial uptake of the flg22 peptide by measuring fluorescence observed in the membrane and vesicles in images of leaf discs floated on TAMRA–flg22 (confocal microscopy) and FAM–flg22 (epifluorescence microscopy). flg22 binding/uptake during the first 1.5 h was reduced in the *fls2* mutant compared with Col and was similar to binding/uptake of scrambled peptide in Col (Fig. 6A, B and Supplementary Fig. S9A). In this initial period, TAMRA–flg22 fluorescence in *bak1* (which is not affected in flg22 binding; Chinchilla *et al.*, 2007) was similar to Col (Fig. 6A, B and Supplementary Fig. S9A). Adding chemicals that are known to effectively inhibit FLS2 internalization (MG132 and 2,3-butanedione monoxime (BDM); Robatzek *et al.*, 2006; Beck *et al.*, 2012) simultaneously with TAMRA–flg22, did not prevent initial peptide binding/uptake (Fig. 6A, B). However, after overnight incubation, TAMRA–flg22 did not accumulate in vacuoles of Col treated with MG132 and BDM or *fls2* and *bak1* without the inhibitors (Fig. 6C, D and Supplementary Fig. S9B).

Uptake/binding during 1 h and overnight accumulation of TAMRA–flg22 were outcompeted by an excess of unlabeled flg22, indicating that these processes require and are limited by FLS2 (Supplementary Fig. S9C, D).

Collectively, our data show that flg22 accumulation in the cells requires RME.

### Long-distance transport of flg22 requires endocytosis

As FLS2-dependent internalization and sustained uptake/accumulation of flg22 in plant cells correlated with long-distance mobility, we hypothesized that endocytosis also plays a role in long-distance transport. Indeed, long-distance movement of TAMRA–flg22 was prevented by inhibitors that affect internalization, MG132 and BDM, in a manner similar to that observed in mutant plants lacking FLS2 or BAK1 coreceptors (Fig. 2A, B).

Our experiments show that flg22 accumulation within cells and long-distance transport within plants require internalization via RME, and binding to the receptor alone is not sufficient for accumulation or movement.

### Defense activation does not contribute to peptide uptake, accumulation and transport

It seemed possible that transport or uptake of phloem-mobile molecules was affected by defense activation due to perception of flg22. Despite reports that activation of immunity reduces a vascular flow and molecular flux through plasmodesmata (Freeman and Beattie, 2009; Oh *et al.*, 2010; Faulkner *et al.*, 2013; Wang *et al.*, 2013), we observed more transport of flg22 than inactive scrambled peptide (Figs 1A and 3A, B). To test if defense induction by flg22 affected general phloem uptake and bulk flow, we evaluated the movement of [^14^C]sucrose in the presence of flg22. Neither the uptake nor the flow was significantly affected by flg22 in our conditions (see Supplementary Fig. S10).

**Fig. 7.**
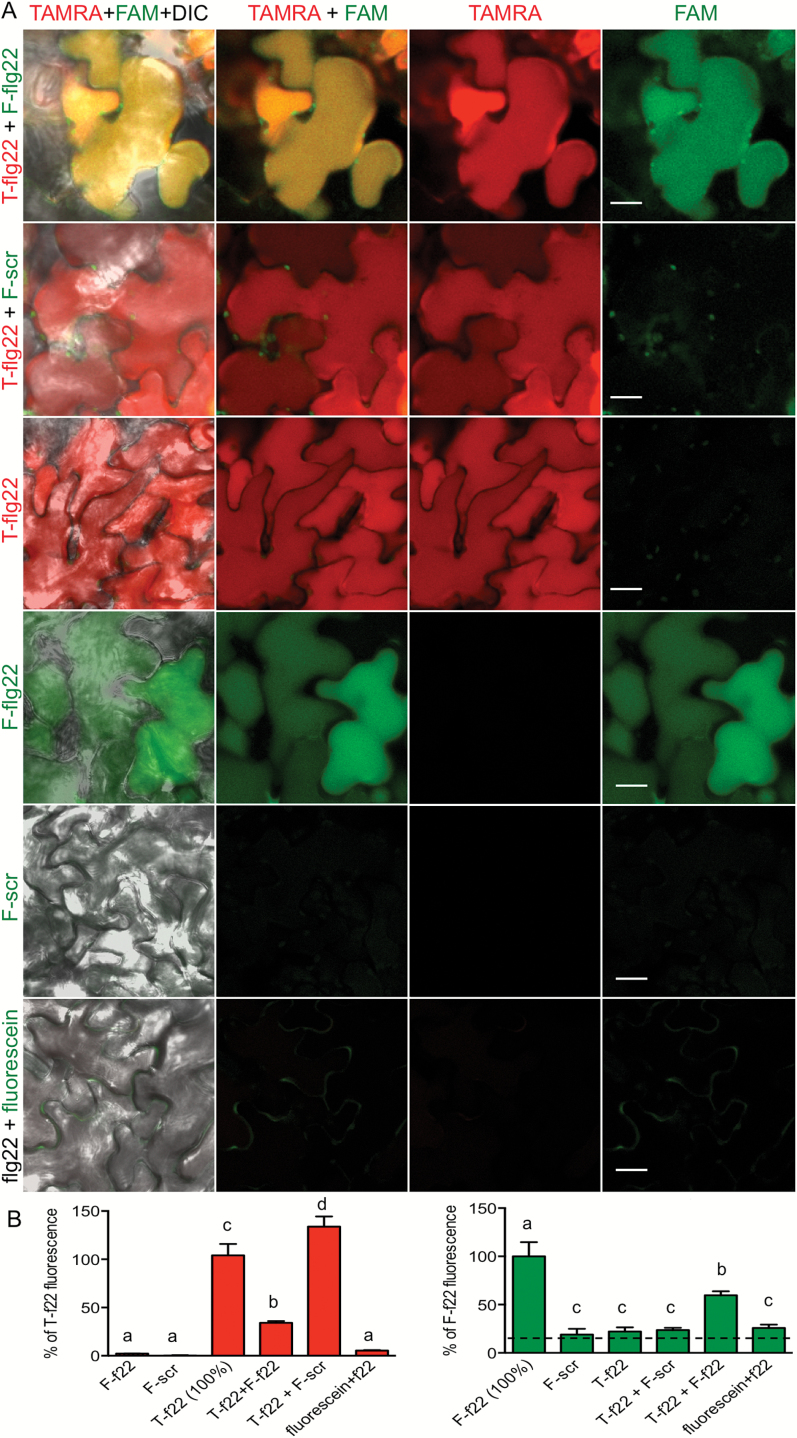
Defense activation does not increase peptide accumulation. Only the active peptide accumulates in Col cells. Defense activation does not make cells permeable to inactive peptide or dye. Leaf discs were floated on indicated mixed peptide solution overnight; 1 μM TAMRA–flg22 (T-flg22), 2 μM FAM–flg22 (F-flg22), 2 μM FAM–scrambled peptide (F-scr), 2 μM flg22 and 2 μM fluorescein were used. (A) Confocal microscopy images in red (TAMRA) and green (FAM, fluorescein) channels and a composite of fluorescence channels with or without DIC are shown. (B) TAMRA and FAM fluorescence was quantified in confocal microscopy images of the same field. Data from at least three experiments per treatment were combined and presented as percentage of fluorescence in leaf discs treated with active peptide alone. Letters show significant difference (*P*<0.05, ANOVA/Tukey’s test, *n*≥8). Dashed line is background fluorescence (mock treatment without peptides). Mixed active peptides show lower fluorescence than single peptide in each channel due to competition of TAMRA–flg22 (T-f22) and FAM–flg22 (F-f22). f22 is flg22.

To test if non-specific peptide uptake and accumulation were affected by defense activation, we floated Col leaf discs on a mixture of active and scrambled peptides labeled with different fluorophores. Only active flg22 accumulated in plant cells and non-specific peptide uptake was not observed, as shown by quantification of TAMRA–flg22 and FAM–scrambled peptide fluorescence in confocal microscopy images (Fig. 7A, B). Detection of both TAMRA–flg22 and FAM–flg22 in the same cells confirmed that both fluorophores could be detected simultaneously (Fig. 7A). In addition, flg22 did not induce uptake of the small molecule fluorescein (Fig. 7) and did not increase uptake or accumulation of TAMRA–flg22, but outcompeted the labeled peptide (see Supplementary Fig. S9C, D).

**Fig. 8.**
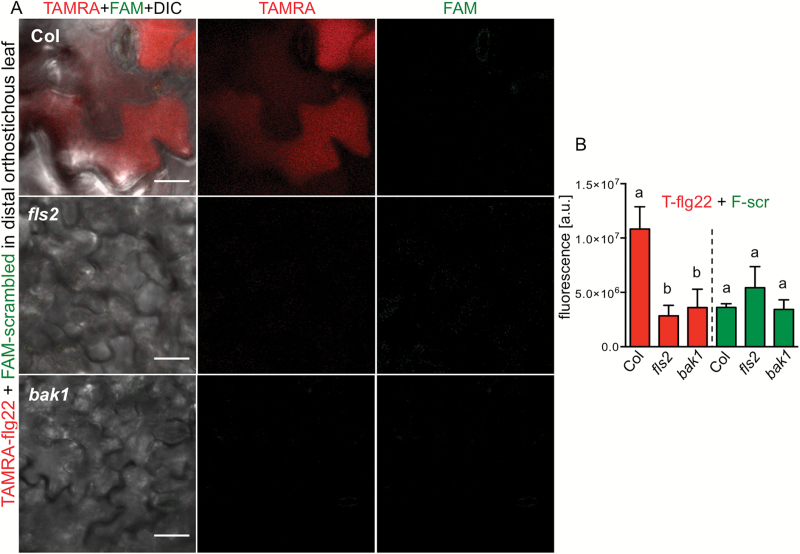
Defense activation does not increase peptide transport. Only active TAMRA–flg22 (5 μM) moves to distal tissue in Col when mixed with FAM–scrambled peptide (10 μM). Peptide transport was not observed in *fls2* and *bak1*. Mixed peptides were applied on a filter paper disc placed on one leaf of *in vitro*-grown plants. (A) Confocal microscopy images of distal orthostichous leaves in red and green channels and a composite of fluorescence channels with DIC are shown. (B) TAMRA and FAM fluorescence was quantified in red and green channels in the same confocal microscopy images of distal orthostichous tissues 1 d after application. Letters indicate significant difference (calculated separately for TAMRA and FAM; *P*<0.05, ANOVA/Tukey’s test, *n*=6).

To further confirm specificity of flg22 transport, we applied a mixture of TAMRA–flg22 and FAM–scrambled peptides on a filter paper disc to one leaf of Arabidopsis. Only TAMRA–flg22 and not FAM–scrambled peptide moved, to a significant extent, to distal organs in WT Col, and neither of the mixed peptides was significantly mobile in *fls2* and *bak1* mutants (Fig. 8). Moreover, both inhibitors that affect receptor internalization, MG132 and BDM, prevented flg22 accumulation and transport (Figs 2 and 6), whereas only BDM impaired defense activation as measured by ROS production (see Supplementary Fig. S1B).

These experiments show that defense activation by flg22 does not induce non-specific transport or uptake of peptides and other phloem-mobile molecules.

## Discussion

flg22–FLS2 is one of the most studied ligand–receptor pairs in plants. Binding of the ligand induces receptor endocytosis, starting a signaling cascade to trigger local defense and SAR (Zipfel *et al.*, 2004; Robatzek *et al.*, 2006; Mishina and Zeier, 2007). We determined the fate of flg22 in cells and whole plants: flg22 accumulates within cells after internalization and is transported to vascular regions and orthostichous distal organs. Endocytosis together with the FLS2 receptor and trafficking along the endocytic pathway typical for plasma membrane proteins (Scheuring *et al.*, 2012), followed by accumulation of flg22 inside the cells, is correlated with long-distance peptide transport. flg22 uptake and transport are specific, as inactive scrambled peptide is not internalized and therefore does not move efficiently. Additionally, activation of defenses *per se* does not contribute to accumulation and transport of flg22. That flg22 can move in plants is not surprising, as long-distance transport was shown for several foreign proteins and peptides (Imlau *et al.*, 1999; Niu *et al.*, 2011). It was surprising, however, that FLS2 is required for efficient transport. Binding to the receptor is not sufficient, because lack of BAK1 coreceptor or disruption of endocytosis prevent flg22 accumulation in cells and long-distance transport. We propose that receptor-mediated endocytosis contributes to flg22 systemic movement by providing access to the cell interior and vascular tissue.

It is rarely recognized that extracellular long-distance signaling molecules must enter cells in order to gain access to phloem. The apoplast is not continuous with phloem (De Schepper *et al.*, 2013). Transporters are required for delivery of many mobile molecules, which otherwise cannot cross membranes, into companion and sieve element cells, either from other surrounding cells (symplastic loading) or from the apoplast (apoplastic loading). For example, glucosinolate membrane transporters, essential for long-distance movement of these defense compounds to seeds, were proposed to control their loading from the apoplast into the phloem (Nour-Eldin *et al.*, 2012). Sugar transporters are necessary for sucrose uptake and movement (De Schepper *et al.*, 2013; Kühn and Grof, 2010). Even micronutrients (minerals) are uploaded into xylem and phloem via specific transporters (reviewed in Lucas *et al.*, 2013).

Our results show that receptor-mediated internalization may be used instead of plasma membrane transporters for delivery of extracellular molecules to the cells and the resulting short- and long-distance trafficking. We detected flg22 in epidermal cells, mesophyll cells and vascular tissue. FLS2 is highly expressed in vasculature and stems (Beck *et al.*, 2014; Genevestigator, www.genevestigator.com, last accessed 6 April 2017; AtGenExpress, http://jsp.weigelworld.org/expviz/expviz.jsp, last accessed 6 April 2017;Schmid *et al.*, 2005) providing entry points for flg22 to phloem cells and long-distance transport. FLS2 is also present in plasmodesmata (Faulkner *et al.*, 2013), where it may contribute to flg22 movement between cells and uploading to phloem. It remains to be established whether the receptor is needed only for the initial uptake or also for further transport along vasculature. Sucrose transporters are present not only in collection phloem (source), but also in transport and release phloem, and it was proposed that leakage (release) and retrieval mechanisms contribute to movement of assimilates along transport phloem (De Schepper *et al.*, 2013).

We do not know by what mechanism flg22 leaves the endocytic pathway to access vascular tissue and long distance routes or if or how it dissociates from FLS2. The fate of flg22 inside plant cells is similar to that of *Potato mop-top* and *Cauliflower mosaic virus* movement proteins, which are endocytosed and delivered to pre-vacuoles/late endosomal structures (Haupt *et al.*, 2005; Carluccio *et al.*, 2014). Endosomal trafficking of the movement proteins is essential for the spread of viruses to distal tissues, as is endocytosis of flg22 for efficient peptide movement to systemic tissue, although it is not clear how movement proteins escape the default pathway and degradation in vacuoles. Many viruses replicate near endomembrane systems and move between cells through plasmodesmata, which they reach by endosomal recycling, secretory pathways or along the cytoskeleton (Schoelz *et al.*, 2011). It is still unknown how viruses enter and exit vascular tissue in apoplastic loading plants such as Arabidopsis (Vuorinen *et al.*, 2011) and in many cases it is not well understood in which form they move within phloem (Hipper *et al.*, 2013). A portion of the flg22 pool may escape the pathway to vacuole and travel in endosomal compartments, as some viral movement proteins do. A mechanism similar to fragmentation and release of flagellin peptides from the TLR5 receptor (flagellin receptor in human cells) for antigen presentation by MHCII (Letran *et al.*, 2011) may exist in plants. Although a previous study showed almost irreversible binding of flg22 to tomato cells and membranes, with very little release of labeled peptide to the medium (outside of cells), it did not address potential release inside the cells, i.e. in vacuoles or elsewhere in the endocytic pathway (Meindl *et al.*, 2000). Whereas binding of flg22 to intact Arabidopsis cells was also irreversible, binding to microsomal membranes was reversible (Bauer *et al.*, 2001); therefore, prior evidence for the release of flg22 from Arabidopsis FLS2 exists.

Metabolites delivered through the vasculature are often released to the apoplast at their final destination by mechanisms similar to uploading in source tissues (Lough and Lucas, 2006; De Schepper *et al.*, 2013), although details of unloading are not fully understood (Dinant and Lemoine, 2010). It was speculated that LPS, which was detected in distal tissues after a local application, might directly contribute to SAR (Zeidler *et al.*, 2010), although further studies are needed to establish if flg22 or LPS directly induces defenses in distal tissues. Mobile elicitins directly induce necrosis in distal tissues (Ricci *et al.*, 1989; Uhlíková *et al.*, 2016). Interestingly, LPS and elicitin receptors have been recently identified in Arabidopsis and wild potato, respectively (Du *et al.*, 2015; Ranf *et al.*, 2015), and future studies may reveal if they are necessary for the ligand movement.

Plants use many signaling peptides in development and defense such as CLE peptides, defensins and PEPs (reviewed in Murphy *et al.*, 2012; Marmiroli and Maestri, 2014) that activate signaling in target cells by interacting with their specific receptors. Receptor-mediated entry to the cells may contribute to transport of these peptides and other endocytosed molecules that were not considered mobile, to neighboring cells or distal tissues, as in the case of flg22.

## Supplementary data

Supplementary data are available at *JXB* online.

Fig. S1. Labeled flg22 peptides are active.

Fig. S2. flg22 movement is specific and FLS2-dependent.

Fig. S3. flg22 moves in an FLS2-dependent manner along plant orthostichy.

Fig. S4. ^125^I-Y-flg22 is not degraded and moves from the treatment site to distal regions.

Fig. S5. TAMRA–flg22 colocalizes with FLS2–GFP at plasma membrane in *N. benthamiana*.

Fig. S6. TAMRA–flg22 colocalizes with FLS2–GFP at plasma membrane in Arabidopsis.

Fig. S7. TAMRA–flg22 is internalized and colocalizes with FLS2–GFP in vesicles.

Fig. S8. TAMRA–flg22 accumulates also in mesophyll cells.

Fig. S9. Accumulation of flg22 in plant cells requires the FLS2 receptor.

Fig. S10. Defense activation does not increase bulk phloem flow.

Table S1. Peptides used in this study.

## Supplementary Material

supplementary_table_S1_figures_S1_S10

## Abbreviations:

BAK1: BRI1-ASSOCIATED KINASE 1, AT4G33430
BDM: 2,3-butanedione monoxime
BRI1: BRASSINOSTEROID INSENSITIVE 1, AT4G39400
DIC: differential interference contrast
FAM: 5-carboxyfluorescein
FLS2: FLAGELLIN SENSITIVE 2, AT5G46330
LPS: lipopolysaccharide
MVB: multivesicular bodies
PAMP: pathogen associated molecular pattern
RME: receptor-mediated endocytosis
ROS: reactive oxygen species
SAR: systemic acquired resistance
TAMRA: 5-carboxytetramethylrhodamine
TGN: trans-Golgi network.

## References

[CIT0001] Albert M 2013 Peptides as triggers of plant defence. Journal of Experimental Botany 64, 5269–5279. 24014869 10.1093/jxb/ert275

[CIT0002] Bauer Z Gómez-Gómez L Boller T Felix G 2001 Sensitivity of different ecotypes and mutants of *Arabidopsis thaliana* toward the bacterial elicitor flagellin correlates with the presence of receptor-binding sites. The Journal of Biological Chemistry 276, 45669–45676. 11564731 10.1074/jbc.M102390200

[CIT0003] Beck M Wyrsch I Strutt J Wimalasekera R Webb A Boller T Robatzek S 2014 Expression patterns of FLAGELLIN SENSING 2 map to bacterial entry sites in plant shoots and roots. Journal of Experimental Botany 65, 6487–6498. 25205577 10.1093/jxb/eru366 PMC4246182

[CIT0004] Beck M Zhou J Faulkner C MacLean D Robatzek S 2012 Spatio-temporal cellular dynamics of the Arabidopsis flagellin receptor reveal activation status-dependent endosomal sorting. The Plant Cell 24, 4205–4219. 23085733 10.1105/tpc.112.100263 PMC3516521

[CIT0005] Ben Khaled S Postma J Robatzek S 2015 A moving view: subcellular trafficking processes in pattern recognition receptor-triggered plant immunity. Annual Review of Phytopathology 53, 379–402. 10.1146/annurev-phyto-080614-120347 26243727

[CIT0006] Bolte S Talbot C Boutte Y Catrice O Read ND Satiat-Jeunemaitre B 2004 FM-dyes as experimental probes for dissecting vesicle trafficking in living plant cells. Journal of Microscopy 214, 159–173. 15102063 10.1111/j.0022-2720.2004.01348.x

[CIT0007] Bozkurt TO Belhaj K Dagdas YF Chaparro-Garcia A Wu CH Cano LM Kamoun S 2015 Rerouting of plant late endocytic trafficking toward a pathogen interface. Traffic 16, 204–226. 25430691 10.1111/tra.12245

[CIT0008] Brauer D Otto J Tu SI 1995 Selective accumulation of the fluorescent Ph indicator, BCECF, in vacuoles of maize root-hair cells. Journal of Plant Physiology 145, 57–61.

[CIT0009] Brummer M Arend M Fromm J Schlenzig A Osswald WF 2002 Ultrastructural changes and immunocytochemical localization of the elicitin quercinin in *Quercus robur* L. roots infected with *Phytophthora quercina*. Physiological and Molecular Plant Pathology 61, 109–120.

[CIT0010] Carluccio AV Zicca S Stavolone L 2014 Hitching a ride on vesicles: cauliflower mosaic virus movement protein trafficking in the endomembrane system. Plant Physiology 164, 1261–1270. 24477592 10.1104/pp.113.234534 PMC3938618

[CIT0011] Chaparro-Garcia A Schwizer S Sklenar J 2015 *Phytophthora infestans* RXLR-WY effector AVR3a associates with DYNAMIN-RELATED PROTEIN 2 required for endocytosis of the plant pattern recognition receptor FLS2. PLoS ONE 10, e0137071. 26348328 10.1371/journal.pone.0137071 PMC4562647

[CIT0012] Chinchilla D Bauer Z Regenass M Boller T Felix G 2006 The Arabidopsis receptor kinase FLS2 binds flg22 and determines the specificity of flagellin perception. The Plant Cell 18, 465–476. 16377758 10.1105/tpc.105.036574 PMC1356552

[CIT0013] Chinchilla D Zipfel C Robatzek S Kemmerling B Nürnberger T Jones JD Felix G Boller T 2007 A flagellin-induced complex of the receptor FLS2 and BAK1 initiates plant defence. Nature 448, 497–500. 17625569 10.1038/nature05999

[CIT0014] Choi SW Tamaki T Ebine K Uemura T Ueda T Nakano A 2013 RABA members act in distinct steps of subcellular trafficking of the FLAGELLIN SENSING2 receptor. The Plant Cell 25, 1174–1187. 23532067 10.1105/tpc.112.108803 PMC3634684

[CIT0015] De Schepper V De Swaef T Bauweraerts I Steppe K 2013 Phloem transport: a review of mechanisms and controls. Journal of Experimental Botany 64, 4839–4850. 24106290 10.1093/jxb/ert302

[CIT0016] Devergne JC Bonnet P Panabières F Blein JP Ricci P 1992 Migration of the fungal protein cryptogein within tobacco plants. Plant Physiology 99, 843–847. 16669010 10.1104/pp.99.3.843 PMC1080554

[CIT0017] Dinant S Lemoine R 2010 The phloem pathway: new issues and old debates. Comptes Rendus Biologies 333, 307–319. 20371105 10.1016/j.crvi.2010.01.006

[CIT0018] Du J Verzaux E Chaparro-Garcia A 2015 Elicitin recognition confers enhanced resistance to *Phytophthora infestans* in potato. Nature Plants 1, 15034. 27247034 10.1038/nplants.2015.34

[CIT0019] Eaves-Pyles T Bu HF Tan XD Cong Y Patel J Davey RA Strasser JE 2011 Luminal-applied flagellin is internalized by polarized intestinal epithelial cells and elicits immune responses via the TLR5 dependent mechanism. PLoS ONE 6, e24869. 21949773 10.1371/journal.pone.0024869 PMC3174220

[CIT0020] Faoro F Maffi D Cantu D Iriti M 2008 Chemical-induced resistance against powdery mildew in barley: the effects of chitosan and benzothiadiazole. Biocontrol 53, 387–401.

[CIT0021] Faulkner C Petutschnig E Benitez-Alfonso Y Beck M Robatzek S Lipka V Maule AJ 2013 LYM2-dependent chitin perception limits molecular flux via plasmodesmata. Proceedings of the National Academy of Sciences of the United States of America 110, 9166–9170. 23674687 10.1073/pnas.1203458110 PMC3670346

[CIT0022] Felix G Duran JD Volko S Boller T 1999 Plants have a sensitive perception system for the most conserved domain of bacterial flagellin. The Plant Journal 18, 265–276. 10377992 10.1046/j.1365-313x.1999.00265.x

[CIT0023] Ferrieri AP Appel HM Schultz JC 2015 Plant vascular architecture determines the pattern of herbivore-induced systemic responses in *Arabidopsis thaliana*. PLoS ONE 10, e0123899. 25879926 10.1371/journal.pone.0123899 PMC4399992

[CIT0024] Freeman BC Beattie GA 2009 Bacterial growth restriction during host resistance to *Pseudomonas syringae* is associated with leaf water loss and localized cessation of vascular activity in *Arabidopsis thaliana*. Molecular Plant-Microbe Interactions 22, 857–867. 19522568 10.1094/MPMI-22-7-0857

[CIT0025] Gómez-Gómez L Boller T 2000 FLS2: an LRR receptor-like kinase involved in the perception of the bacterial elicitor flagellin in Arabidopsis. Molecular Cell 5, 1003–1011. 10911994 10.1016/s1097-2765(00)80265-8

[CIT0026] Gross A Kapp D Nielsen T Niehaus K 2005 Endocytosis of *Xanthomonas campestris* pathovar *campestris* lipopolysaccharides in non-host plant cells of *Nicotiana tabacum*. The New Phytologist 165, 215–226. 15720635 10.1111/j.1469-8137.2004.01245.x

[CIT0027] Haupt S Cowan GH Ziegler A Roberts AG Oparka KJ Torrance L 2005 Two plant-viral movement proteins traffic in the endocytic recycling pathway. The Plant Cell 17, 164–181. 15608333 10.1105/tpc.104.027821 PMC544497

[CIT0028] Heese A Hann DR Gimenez-Ibanez S Jones AM He K Li J Schroeder JI Peck SC Rathjen JP 2007 The receptor-like kinase SERK3/BAK1 is a central regulator of innate immunity in plants. Proceedings of the National Academy of Sciences of the United States of America 104, 12217–12222. 17626179 10.1073/pnas.0705306104 PMC1924592

[CIT0029] Hipper C Brault V Ziegler-Graff V Revers F 2013 Viral and cellular factors involved in phloem transport of plant viruses. Frontiers in Plant Science 4, 154. 23745125 10.3389/fpls.2013.00154 PMC3662875

[CIT0030] Hunter PR Craddock CP Di Benedetto S Roberts LM Frigerio L 2007 Fluorescent reporter proteins for the tonoplast and the vacuolar lumen identify a single vacuolar compartment in Arabidopsis cells. Plant Physiology 145, 1371–1382. 17905861 10.1104/pp.107.103945 PMC2151705

[CIT0031] Imlau A Truernit E Sauer N 1999 Cell-to-cell and long-distance trafficking of the green fluorescent protein in the phloem and symplastic unloading of the protein into sink tissues. The Plant Cell 11, 309–322. 10072393 10.1105/tpc.11.3.309 PMC144181

[CIT0032] Irani NG Di Rubbo S Mylle E 2012 Fluorescent castasterone reveals BRI1 signaling from the plasma membrane. Nature Chemical Biology 8, 583–589. 22561410 10.1038/nchembio.958

[CIT0033] Kang Y Jelenska J Cecchini NM Li Y Lee MW Kovar DR Greenberg JT 2014 HopW1 from *Pseudomonas syringae* disrupts the actin cytoskeleton to promote virulence in Arabidopsis. PLoS Pathogens 10, e1004232. 24968323 10.1371/journal.ppat.1004232 PMC4072799

[CIT0034] Keinath NF Waadt R Brugman R Schroeder JI Grossmann G Schumacher K Krebs M 2015 Live cell imaging with R-GECO1 sheds light on flg22- and chitin-induced transient [Ca^2+^]_cyt_ patterns in Arabidopsis. Molecular Plant 8, 1188–1200. 26002145 10.1016/j.molp.2015.05.006 PMC5134422

[CIT0035] Kiefer IW Slusarenko AJ 2003 The pattern of systemic acquired resistance induction within the Arabidopsis rosette in relation to the pattern of translocation. Plant Physiology 132, 840–847. 12805614 10.1104/pp.103.021709 PMC167024

[CIT0036] Kühn C Grof CP 2010 Sucrose transporters of higher plants. Current Opinion in Plant Biology 13, 288–298. 20303321 10.1016/j.pbi.2010.02.001

[CIT0037] Letran SE Lee SJ Atif SM Uematsu S Akira S McSorley SJ 2011 TLR5 functions as an endocytic receptor to enhance flagellin-specific adaptive immunity. European Journal of Immunology 41, 29–38. 21182074 10.1002/eji.201040717 PMC3652676

[CIT0038] Lough TJ Lucas WJ 2006 Integrative plant biology: role of phloem long-distance macromolecular trafficking. Annual Review of Plant Biology 57, 203–232. 10.1146/annurev.arplant.56.032604.144145 16669761

[CIT0039] Lu D Lin W Gao X 2011 Direct ubiquitination of pattern recognition receptor FLS2 attenuates plant innate immunity. Science 332, 1439–1442. 21680842 10.1126/science.1204903 PMC3243913

[CIT0040] Lucas WJ Groover A Lichtenberger R 2013 The plant vascular system: evolution, development and functions. Journal of Integrative Plant Biology 55, 294–388. 23462277 10.1111/jipb.12041

[CIT0041] Marmiroli N Maestri E 2014 Plant peptides in defense and signaling. Peptides 56, 30–44. 24681437 10.1016/j.peptides.2014.03.013

[CIT0042] Martins S Dohmann EM Cayrel A 2015 Internalization and vacuolar targeting of the brassinosteroid hormone receptor BRI1 are regulated by ubiquitination. Nature Communications 6, 6151. 10.1038/ncomms7151 PMC4713032 25608221

[CIT0043] McConahey PJ Dixon FJ 1966 A method of trace iodination of proteins for immunologic studies. International Archives of Allergy and Applied Immunology 29, 185–189. 4160044 10.1159/000229699

[CIT0044] Meindl T Boller T Felix G 2000 The bacterial elicitor flagellin activates its receptor in tomato cells according to the address-message concept. The Plant Cell 12, 1783–1794. 11006347 10.1105/tpc.12.9.1783 PMC149085

[CIT0045] Mishina TE Zeier J 2007 Pathogen-associated molecular pattern recognition rather than development of tissue necrosis contributes to bacterial induction of systemic acquired resistance in Arabidopsis. The Plant Journal 50, 500–513. 17419843 10.1111/j.1365-313X.2007.03067.x

[CIT0046] Mott GA Middleton MA Desveaux D Guttman DS 2014 Peptides and small molecules of the plant-pathogen apoplastic arena. Frontiers in Plant Science 5, 677. 25506352 10.3389/fpls.2014.00677 PMC4246658

[CIT0047] Murphy E Smith S De Smet I 2012 Small signaling peptides in Arabidopsis development: how cells communicate over a short distance. The Plant Cell 24, 3198–3217. 22932676 10.1105/tpc.112.099010 PMC3462626

[CIT0048] Niu C Smith N Garteiser P Towner R Verchot J 2011 Comparative analysis of protein transport in the *N. benthamiana* vasculature reveals different destinations. Plant Signaling & Behavior 6, 1793–1808. 22057341 10.4161/psb.6.11.17896 PMC3329354

[CIT0049] Nour-Eldin HH Andersen TG Burow M 2012 NRT/PTR transporters are essential for translocation of glucosinolate defence compounds to seeds. Nature 488, 531–534. 22864417 10.1038/nature11285

[CIT0050] Oh HS Park DH Collmer A 2010 Components of the *Pseudomonas syringae* type III secretion system can suppress and may elicit plant innate immunity. Molecular Plant-Microbe Interactions 23, 727–739. 20459312 10.1094/MPMI-23-6-0727

[CIT0051] Ranf S Gisch N Schäffer M 2015 A lectin S-domain receptor kinase mediates lipopolysaccharide sensing in *Arabidopsis thaliana*. Nature Immunology 16, 426–433. 25729922 10.1038/ni.3124

[CIT0052] Ricci P Bonnet P Huet JC Sallantin M Beauvais-Cante F Bruneteau M Billard V Michel G Pernollet JC 1989 Structure and activity of proteins from pathogenic fungi *Phytophthora* eliciting necrosis and acquired resistance in tobacco. European Journal of Biochemistry 183, 555–563. 2776750 10.1111/j.1432-1033.1989.tb21084.x

[CIT0053] Robatzek S Chinchilla D Boller T 2006 Ligand-induced endocytosis of the pattern recognition receptor FLS2 in Arabidopsis. Genes & Development 20, 537–542. 16510871 10.1101/gad.366506 PMC1410809

[CIT0054] Roberts K Love AJ Laval V Laird J Tomos AD Hooks MA Milner JJ 2007 Long-distance movement of *Cauliflower mosaic virus* and host defence responses in Arabidopsis follow a predictable pattern that is determined by the leaf orthostichy. The New Phytologist 175, 707–717. 17688586 10.1111/j.1469-8137.2007.02136.x

[CIT0055] Russinova E Borst JW Kwaaitaal M Caño-Delgado A Yin Y Chory J de Vries SC 2004 Heterodimerization and endocytosis of Arabidopsis brassinosteroid receptors BRI1 and AtSERK3 (BAK1). The Plant Cell 16, 3216–3229. 15548744 10.1105/tpc.104.025387 PMC535869

[CIT0056] Scheuring D Künzl F Viotti C Yan MS Jiang L Schellmann S Robinson DG Pimpl P 2012 Ubiquitin initiates sorting of Golgi and plasma membrane proteins into the vacuolar degradation pathway. BMC Plant Biology 12, 164. 22970698 10.1186/1471-2229-12-164 PMC3534617

[CIT0057] Scheuring D Schöller M Kleine-Vehn J Löfke C 2015 Vacuolar staining methods in plant cells. Methods in Molecular Biology 1242, 83–92. 25408446 10.1007/978-1-4939-1902-4_8

[CIT0058] Schmid M Davison TS Henz SR Pape UJ Demar M Vingron M Schölkopf B Weigel D Lohmann JU 2005 A gene expression map of *Arabidopsis thaliana* development. Nature Genetics 37, 501–506. 15806101 10.1038/ng1543

[CIT0059] Schoelz JE Harries PA Nelson RS 2011 Intracellular transport of plant viruses: finding the door out of the cell. Molecular Plant 4, 813–831. 21896501 10.1093/mp/ssr070 PMC3183398

[CIT0060] Smith JM Salamango DJ Leslie ME Collins CA Heese A 2014 Sensitivity to Flg22 is modulated by ligand-induced degradation and de novo synthesis of the endogenous flagellin-receptor FLAGELLIN-SENSING2. Plant Physiology 164, 440–454. 24220680 10.1104/pp.113.229179 PMC3875820

[CIT0061] Spallek T Beck M Ben Khaled S Salomon S Bourdais G Schellmann S Robatzek S 2013 ESCRT-I mediates FLS2 endosomal sorting and plant immunity. PLoS Genetics 9, e1004035. 24385929 10.1371/journal.pgen.1004035 PMC3873229

[CIT0062] Sun Y Li L Macho AP Han Z Hu Z Zipfel C Zhou JM Chai J 2013 Structural basis for flg22-induced activation of the Arabidopsis FLS2-BAK1 immune complex. Science 342, 624–628. 24114786 10.1126/science.1243825

[CIT0063] Tamura K Shimada T Ono E Tanaka Y Nagatani A Higashi SI Watanabe M Nishimura M Hara-Nishimura I 2003 Why green fluorescent fusion proteins have not been observed in the vacuoles of higher plants. The Plant Journal 35, 545–555. 12904216 10.1046/j.1365-313x.2003.01822.x

[CIT0064] Tateda C Zhang Z Shrestha J Jelenska J Chinchilla D Greenberg JT 2014 Salicylic acid regulates Arabidopsis microbial pattern receptor kinase levels and signaling. The Plant Cell 26, 4171–4187. 25315322 10.1105/tpc.114.131938 PMC4247590

[CIT0065] Thor K Peiter E 2014 Cytosolic calcium signals elicited by the pathogen-associated molecular pattern flg22 in stomatal guard cells are of an oscillatory nature. The New Phytologist 204, 873–881. 25243759 10.1111/nph.13064

[CIT0066] Uhlíková H Obořil M Klempová J Šedo O Zdráhal Z Kašparovský T Skládal P Lochman J 2016 Elicitin-induced distal systemic resistance in plants is mediated through the protein-protein interactions influenced by selected lysine residues. Frontiers in Plant Science 7, 59. 26904041 10.3389/fpls.2016.00059 PMC4742723

[CIT0067] van Gisbergen PA Esseling-Ozdoba A Vos JW 2008 Microinjecting FM4-64 validates it as a marker of the endocytic pathway in plants. Journal of Microscopy 231, 284–290. 18778426 10.1111/j.1365-2818.2008.02041.x

[CIT0068] Vuorinen AL Kelloniemi J Valkonen JP 2011 Why do viruses need phloem for systemic invasion of plants? Plant Science 181, 355–363. 21889041 10.1016/j.plantsci.2011.06.008

[CIT0069] Wang X Sager R Cui W Zhang C Lu H Lee JY 2013 Salicylic acid regulates plasmodesmata closure during innate immune responses in Arabidopsis. The Plant Cell 25, 2315–2329. 23749844 10.1105/tpc.113.110676 PMC3723628

[CIT0070] Zeidler D Dubery IA Schmitt-Kopplin P Von Rad U Durner J 2010 Lipopolysaccharide mobility in leaf tissue of *Arabidopsis thaliana*. Molecular Plant Pathology 11, 747–755. 21029320 10.1111/j.1364-3703.2010.00638.x PMC6640497

[CIT0071] Zhang C Turgeon R 2009 Downregulating the sucrose transporter *VpSUT1* in *Verbascum phoeniceum* does not inhibit phloem loading. Proceedings of the National Academy of Sciences of the United States of America 106, 18849–18854. 19846784 10.1073/pnas.0904189106 PMC2774004

[CIT0072] Zipfel C Robatzek S Navarro L Oakeley EJ Jones JD Felix G Boller T 2004 Bacterial disease resistance in *Arabidopsis* through flagellin perception. Nature 428, 764–767. 15085136 10.1038/nature02485

