# Supplementary material for: Flagellin peptide flg22 gains access to long-distance trafficking in Arabidopsis via its receptor, FLS2
Source: J Exp Bot. 2017 Apr 28;68(7):1769–83. doi: 10.1093/jxb/erx060 (PMC5444442; doi:10.1093/jxb/erx060)
Supplement: supplementary_table_S1_figures_S1_S10 [file erx060_suppl_supplementary_table_S1_figures_S1_S10.pdf]

Name	Sequence
FAM-flg22	5-FAM-QRLSTGSRINSAKDDAAGLQIA
FAM-scrambled	5-FAM-GITASKALGADLRRINQSASQD
FAM-Y-flg22	5-FAM-YQRLSTGSRINSAKDDAAGLQIA
FAM-Y-scrambled	5-FAM-YGITASKALGADLRRINQSASQD
TAMRA-Flg22	TAMRA-QRLSTGSRINSAKDDAAGLQIA
^{125}I-Y-flg22	^{125}I-YQRLSTGSRINSAKDDAAGLQIA
^{125}I-Y-scrambled	^{125}I-YGITASKALGADLRRINQSASQD
flg22	QRLSTGSRINSAKDDAAGLQIA

Supplementary Table S1. Peptides used in this study.

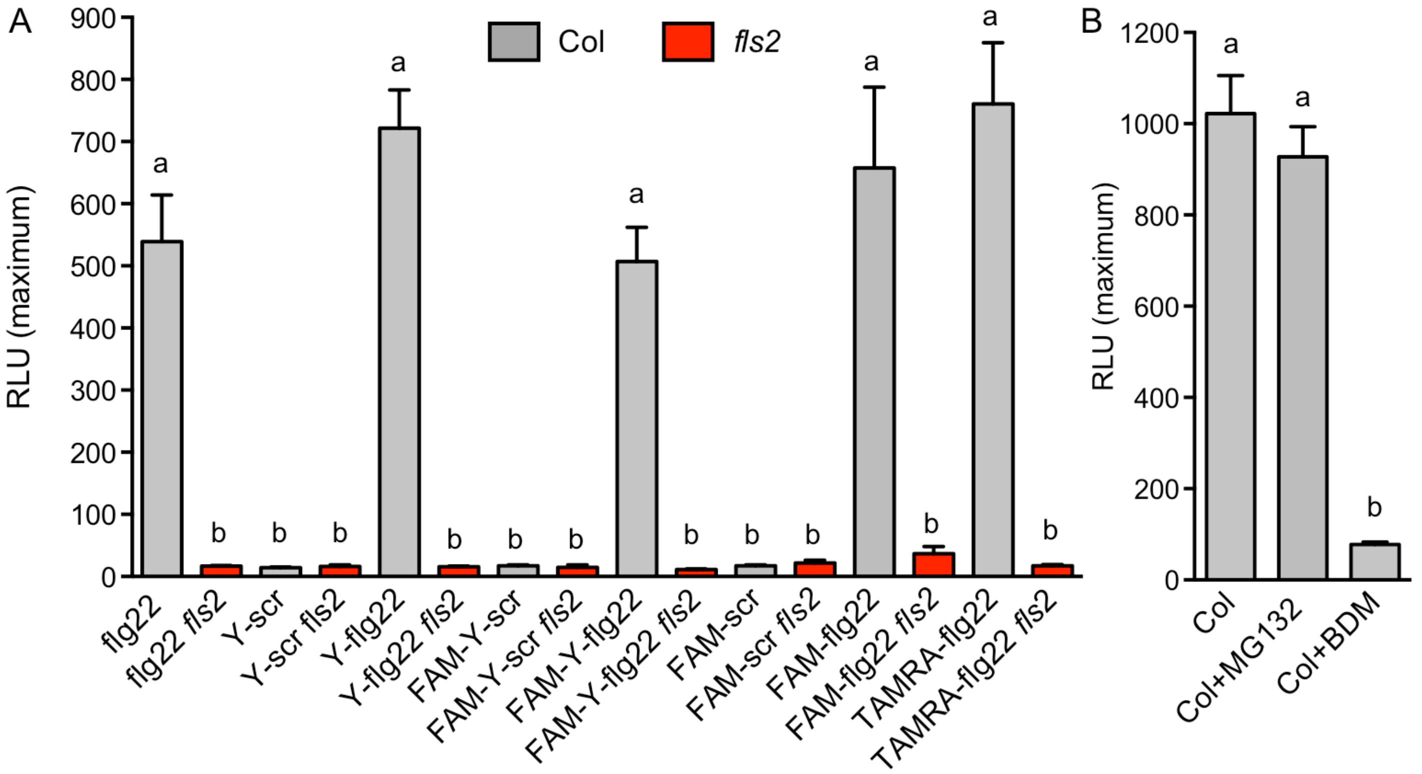

Supplementary Fig. S1. Labeled flg22 peptides are active.
Maximum ROS (with SE) was measured by chemiluminescence after treatment of leaf discs with 1 µM peptides. Letters indicate significant difference (ANOVA/Tukey's test). RLU, relative light units. This experiment was repeated at least twice for each peptide.
A. Labeled flg22 peptides induce ROS in Col, whereas the *fls2* mutant does not respond to peptide treatments. Control scrambled (scr) peptides have no activity. $P<0.002$, $n=8$ for Col and $n=4$ for *fls2*).
B. Endocytosis inhibitors differently affect induction of ROS by flg22. MG132 or BDM were added simultaneously with the peptide. $P<0.0001$, $n=12$.

A

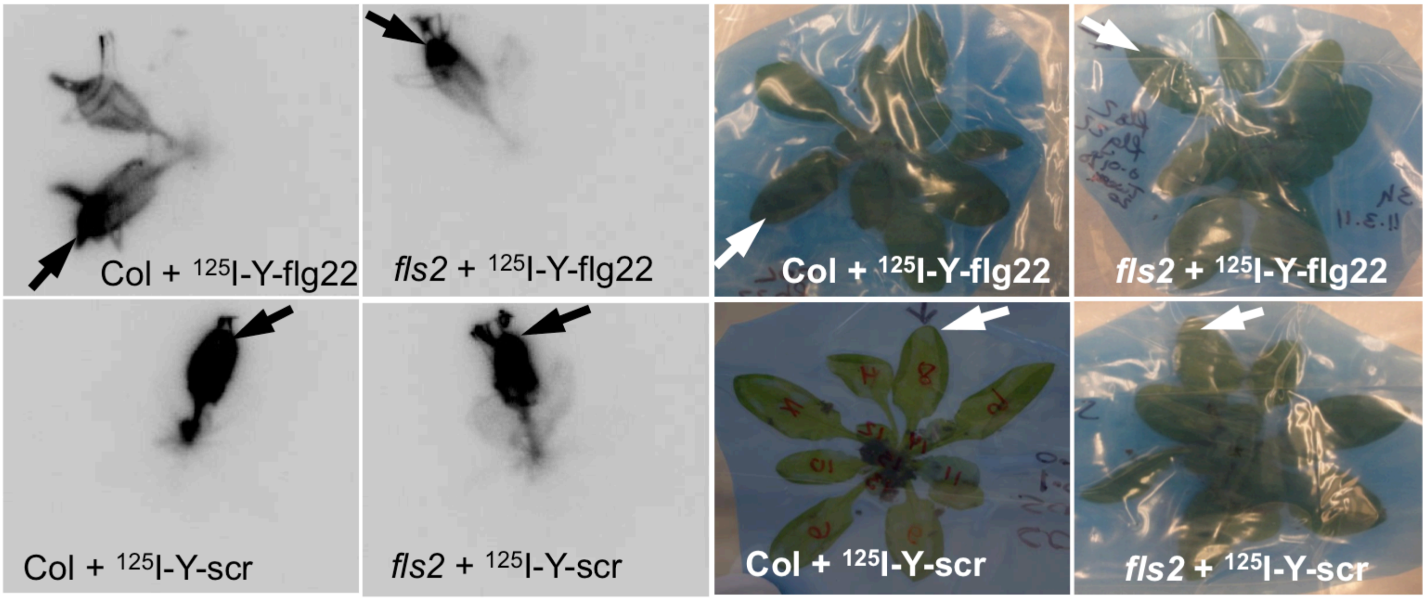

B

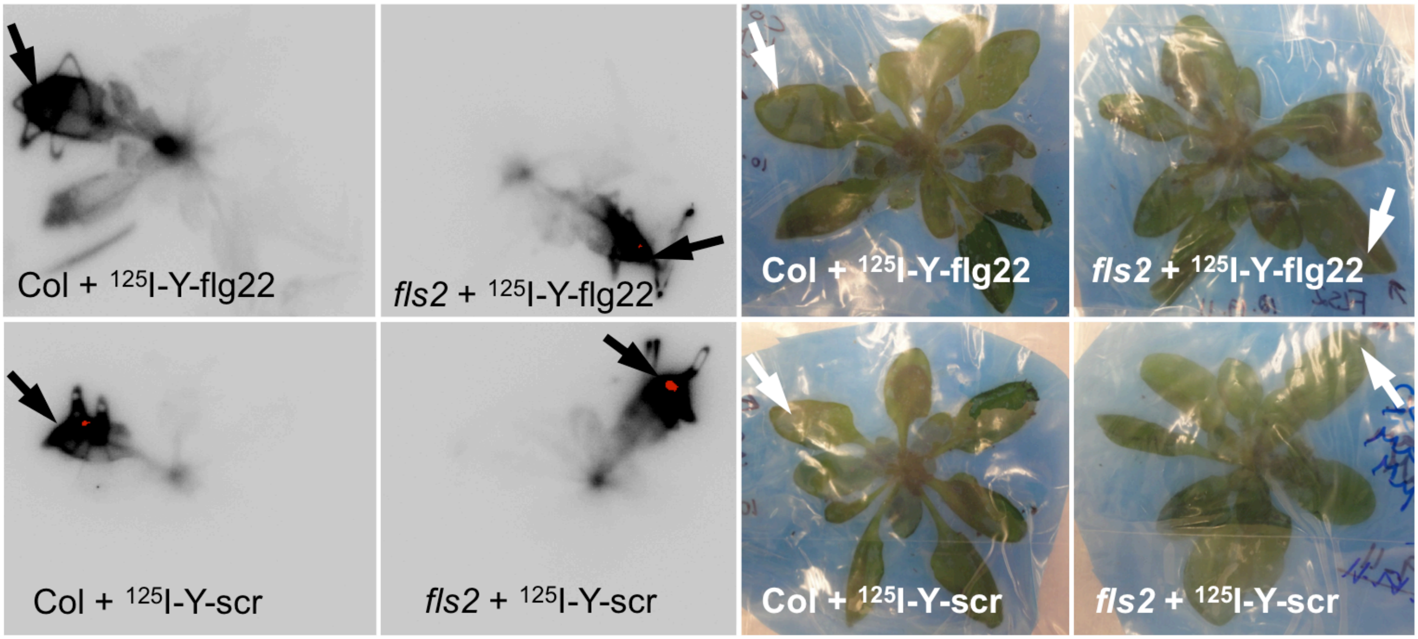

Supplementary Fig. S2. Flg22 movement is specific and FLS2-dependent. ^{125}I-Y-flg22 is detected 3h after application in distal orthostichous leaves of Col but not *fls2*. Scrambled peptide (scr) is not mobile. Plants were treated with radiolabeled peptides as in Fig. 3 [1 µl droplet of 1 µM radiolabeled peptide was applied to the abaxial side of one leaf (arrow) of plants grown in soil and imaged by autoradiography after 3h]. A,B. Images of additional plants from independent experiments are shown.

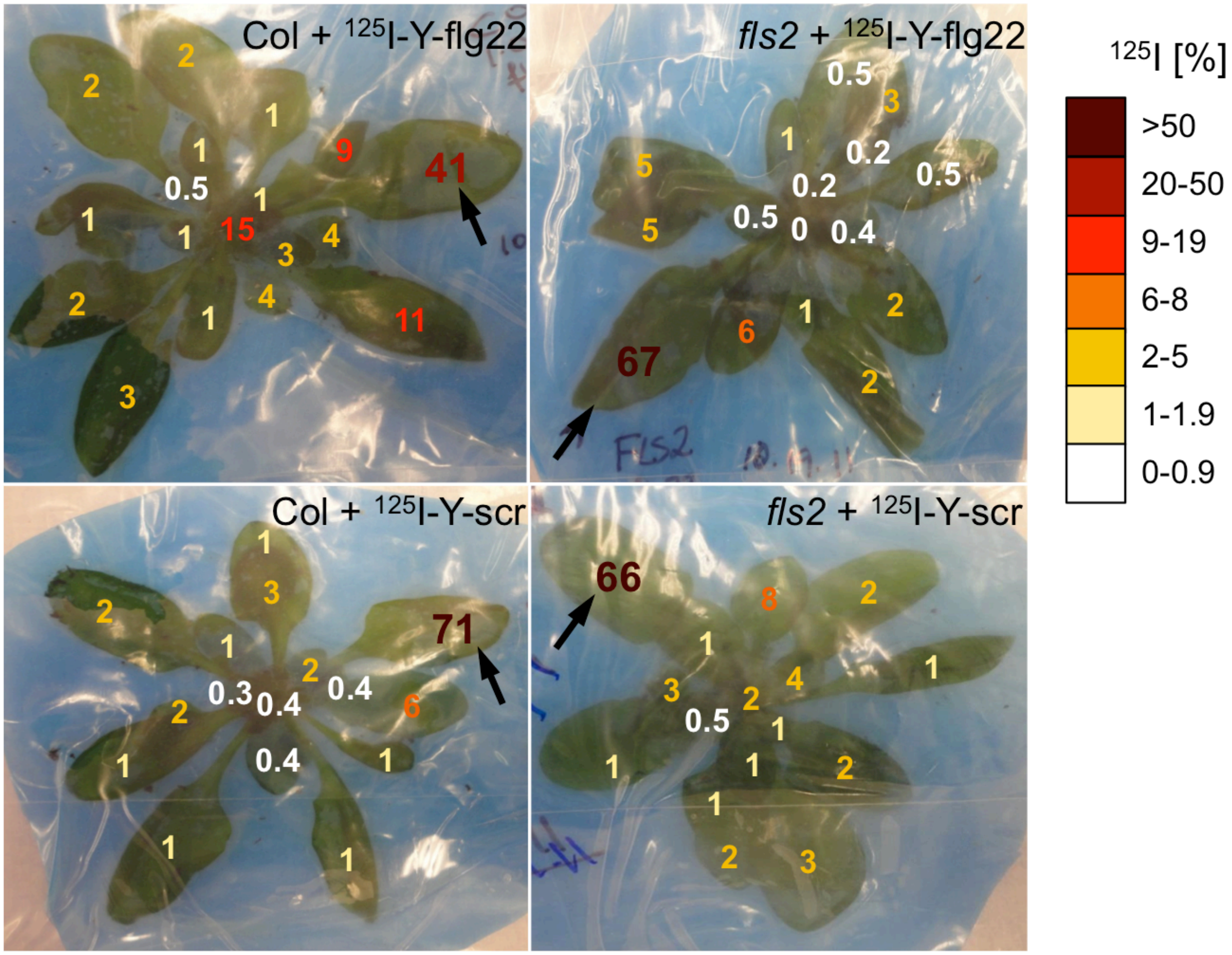

Supplementary Fig. S3. Flg22 moves in a FLS2-dependent manner along plant orthostichy.

1 µl droplet of 0.5 µM radiolabeled peptide was applied on abaxial side of one leaf (arrow) of plants grown in soil. Radioactivity in individual leaves quantified after 3 h by scintillation counting is shown as percentage of total radioactivity in a plant, with highest to lowest signals depicted by darkest to lightest number shades. The highest radioactivity was present in distal Col leaves on the same side of the rosette as ^{125}I-Y-flg22 treated leaf (orthostichous leaves) and in the rosette center.

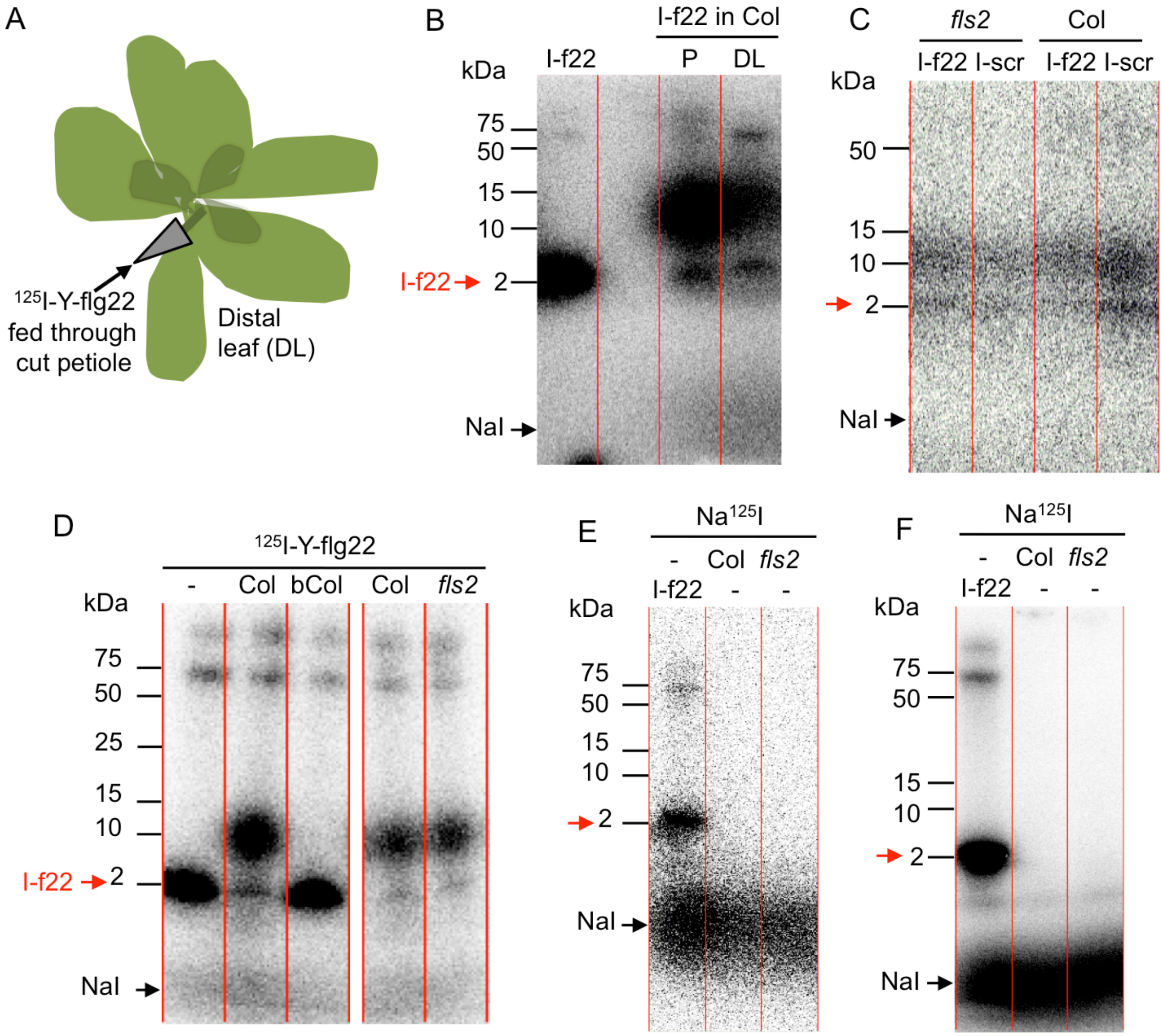

Supplementary Fig. S4. ^{125}I-Y-flg22 is not degraded and moves from the treatment site to distal regions.

Proteins were extracted from indicated tissues, separated by SDS-PAGE and detected by autoradiography. Each experiment was repeated two or more times.

A,B. flg22 is detected in distal leaves of petiole-fed plants. ^{125}I-Y-flg22 was supplied through a tube attached to the cut petiole of a fully expanded Col leaf for 3 h (A). Proteins were extracted from washed petiole and distal orthostichous leaves (B). I-f22, ^{125}I-Y-flg22; P, petiole extract; DL, extract from distal leaf.

C. Size-shift of flg22 peptide is not sequence or receptor dependent. ^{125}I-Y-flg22 and ^{125}I-Y-scrambled (I-scr) were applied to Arabidopsis leaves through petioles for 2 h.

D. Fresh plant extract modifies flg22 to a slower-migrating product independent of FLS2. ^{125}I-Y-flg22 was incubated for 3 h with fresh Col and *fls2* extracts, boiled Col extract (bCol) or alone (-). Parts of the same gel (same exposure) are shown. Boiled extract lost the ability to cause a shift in flg22 migration.

E,F. Na^{125}I does not form a complex with plant proteins. A droplet of Na^{125}I was applied on abaxial side of one Arabidopsis leaf and proteins were extracted from the treated leaf (E). Alternatively, Na^{125}I was incubated with plant extracts for 3 h (F). Standard ^{125}I-Y-flg22 mixed with Na^{125}I is in the first lane of gels in (E,F). Unmodified ^{125}I-Y-flg22 and ^{125}I-Y-scrambled migrate at 2 kDa (red arrow), larger band appears at 12 kDa, and free iodine is indicated by black arrow.

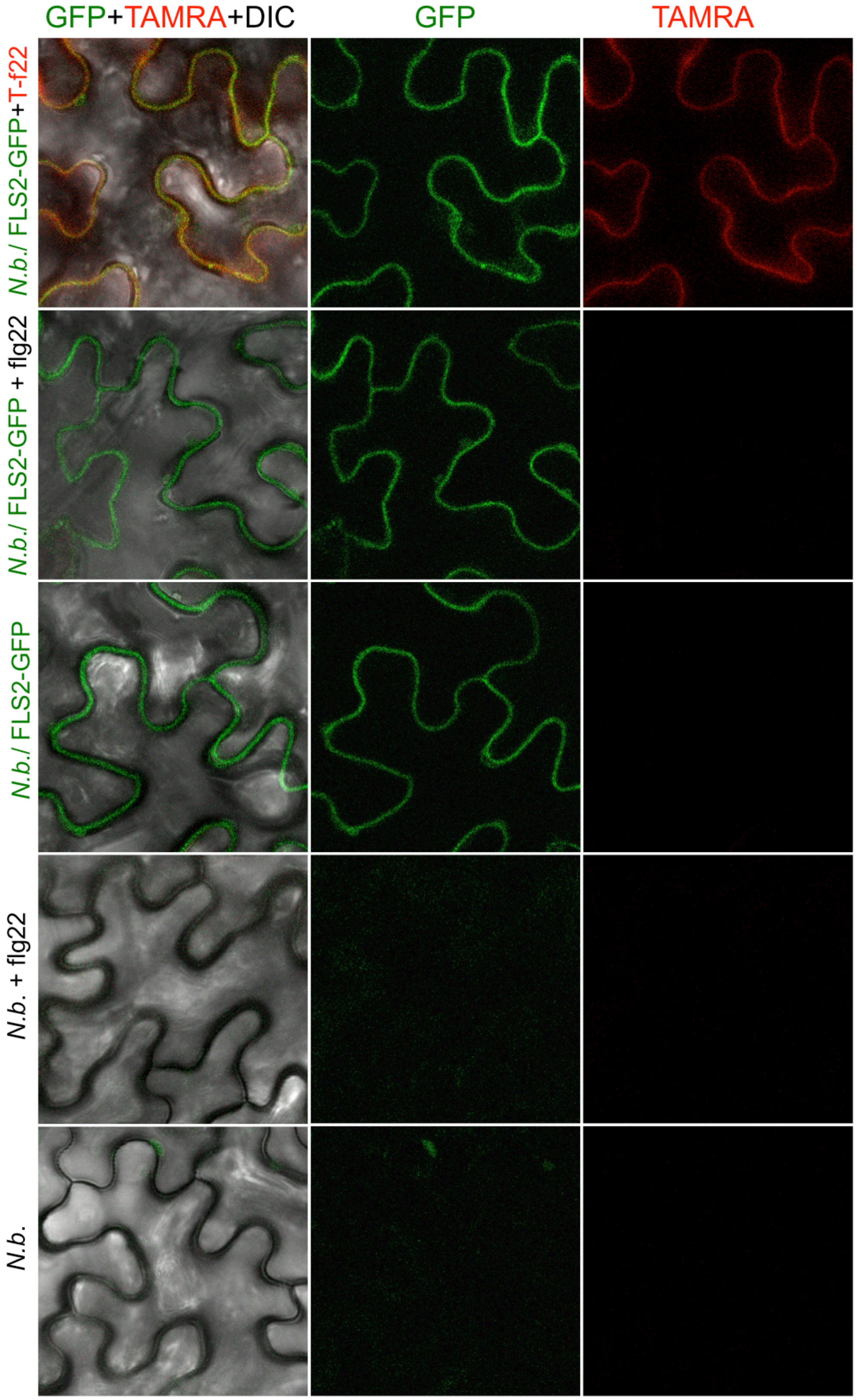

Supplementary Fig. S5. TAMRA-flg22 colocalizes with FLS2-GFP at plasma membrane in *N. benthamiana*. Leaf discs of *N. benthamiana* expressing FLS2-GFP or WT *N. benthamiana* were floated on 2 μM TAMRA-flg22 (Tf22) for 20 min., washed in water and imaged by confocal microscopy. Images in red (TAMRA) and green (GFP) channels, and their composite with DIC image are shown. Unlabeled flg22 does not cause autofluorescence in red or green channels.

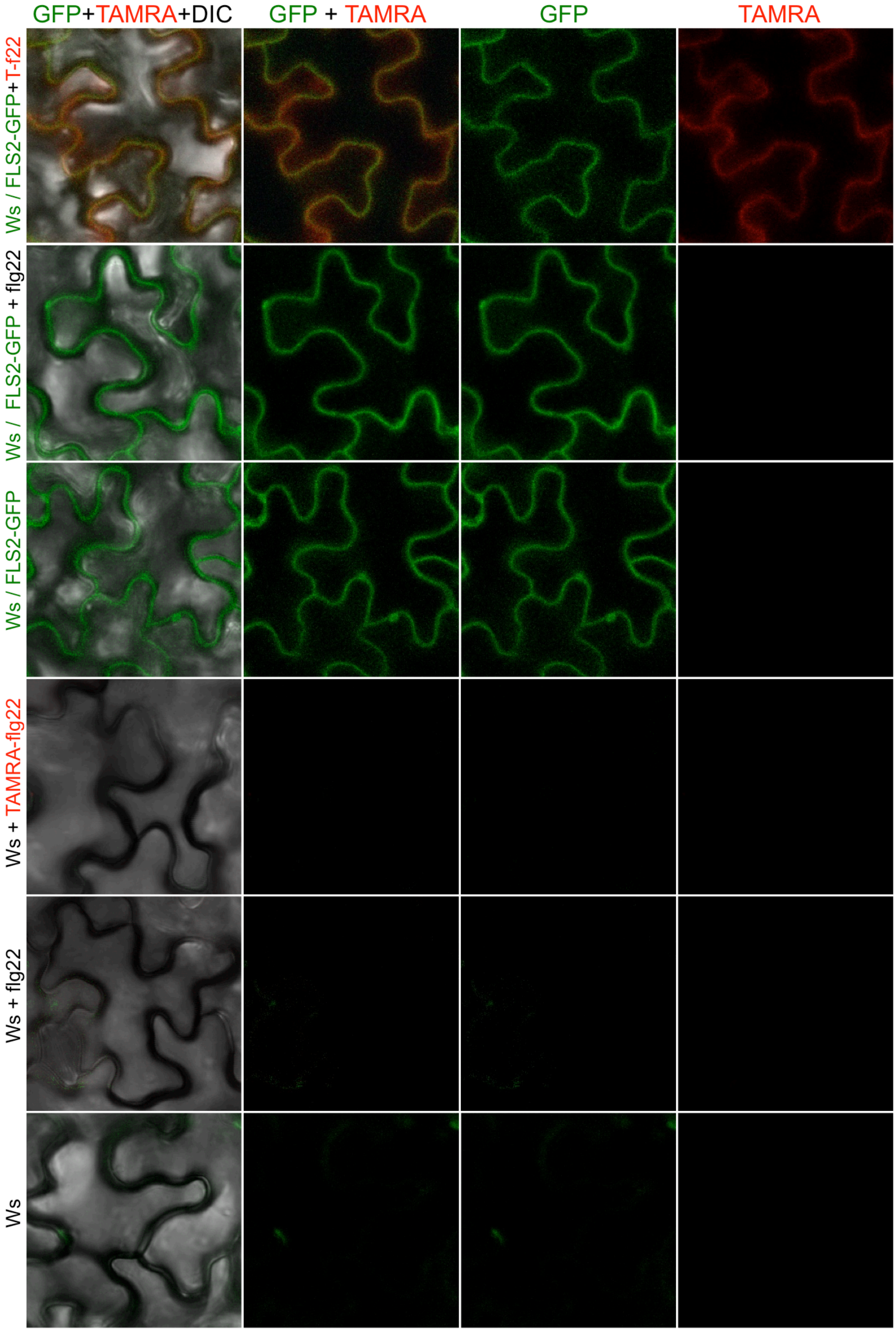

Supplementary Fig. S6.

Supplementary Fig. S6. TAMRA-flg22 colocalizes with FLS2-GFP at plasma membrane in *Arabidopsis*. Leaf discs of *Arabidopsis* Ws expressing FLS2-GFP or WT were floated on 2 μM TAMRA-flg22 (Tf22) for 15-20 min., washed in water and imaged by confocal microscopy. Images in red (TAMRA) and green (GFP) channels, and their composite with or without DIC image are shown. TAMRA-flg22 does not bind to WT Ws plasma membrane. Unlabeled flg22 does not cause autofluorescence in red or green channels.

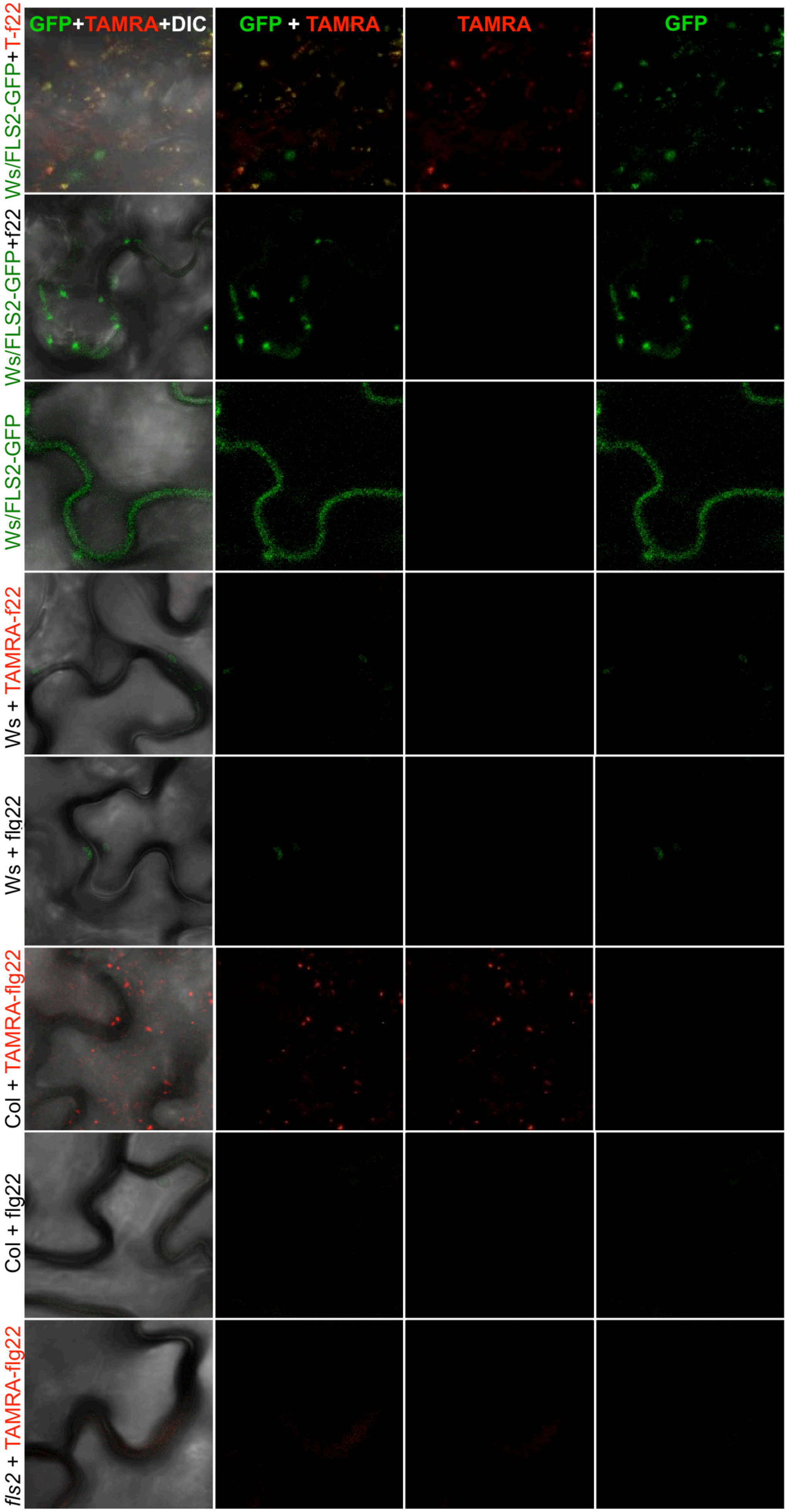

Supplementary Fig. S7.

Supplementary Fig. S7. TAMRA-flg22 is internalized and colocalizes with FLS2-GFP in vesicles. Leaf discs of *Arabidopsis* Ws expressing FLS2-GFP, WT Ws (lacking FLS2) and WT Col (with WT unlabeled FLS2) were floated on 2 µM TAMRA-flg22 (Tf22) for 1-1.5 h, washed in water and imaged by confocal microscopy. Images in red (TAMRA) and green (GFP) channels, and their composite with or without DIC image are shown. TAMRA-flg22 is internalized in Ws/FLS2-GFP and in WT Col. Unlabeled flg22 and TAMRA-flg22 induce internalization of FLS2-GFP. TAMRA-flg22 is not internalized in WT Ws or Col *fls2* that lack FLS2. Unlabeled flg22 does not cause autofluorescence in red or green channels. There is no bleed through signal between green and red channels.

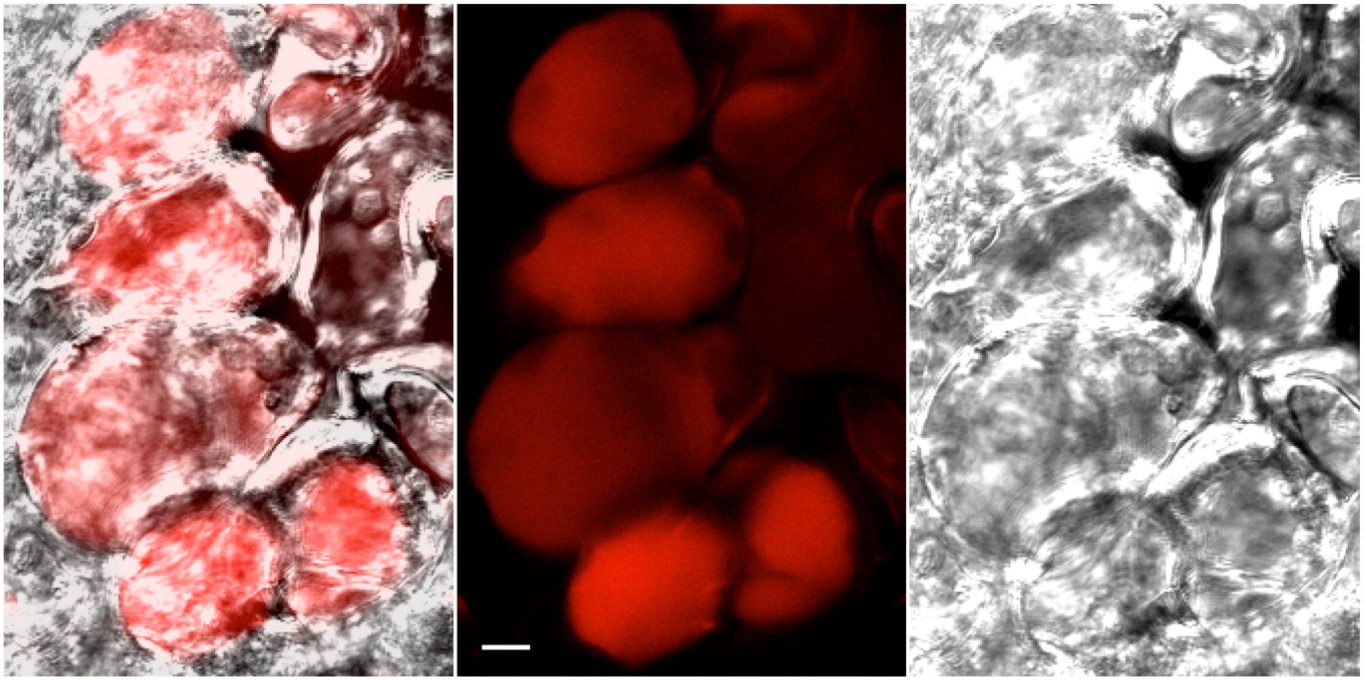

Supplementary Fig. S8. TAMRA-flg22 accumulates also in mesophyll cells. Arabidopsis leaf discs were floated overnight on 1 μM TAMRA-flg22, washed in water and imaged by confocal microscopy. Bar = 10 μm.

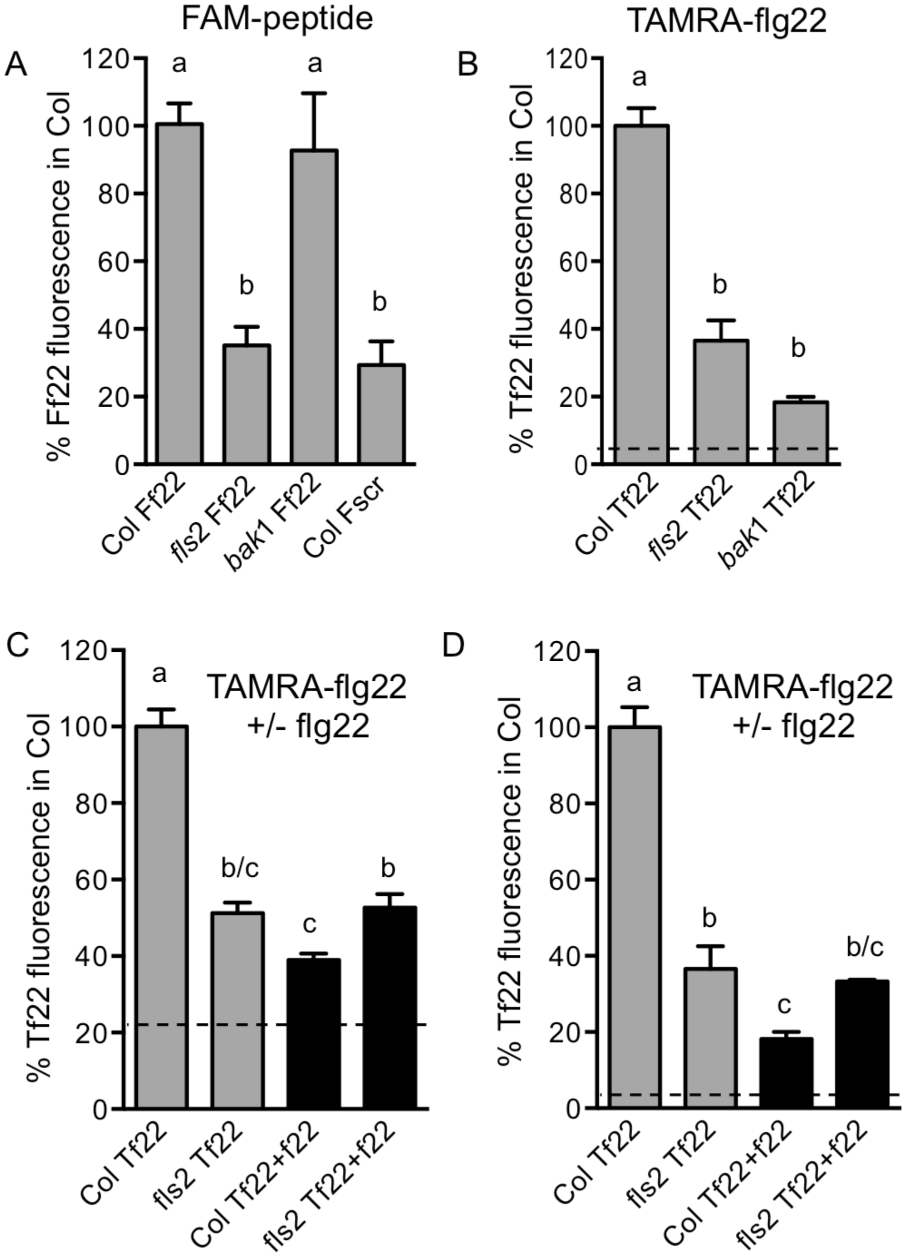

Supplementary Fig. S9. Accumulation of flg22 in plant cells requires the FLS2 receptor. Leaf discs were floated on peptide solution for 1 h (A,C) or overnight (B,D) and fluorescence was quantified in epifluorescence microscopy images from at least 2 experiments. Letters indicate significant difference (ANOVA/Tukey's test). Background fluorescence (mock treatment without peptides) is shown by dashed line.

A. Initial (up to 1 h) uptake/binding of FAM-flg22 (Ff22, 5 μM) is reduced in *fls2* to a similar level as uptake of FAM-scrambled peptide (Fscr), but is not highly affected in *bak1*. $P<0.01$, $n\geq6$. Background fluorescence was subtracted from total fluorescence.

B. Overnight accumulation of TAMRA-flg22 (Tf22, 1 μM) in plant cells is inhibited in *fls2* and *bak1* mutants. $P<0.0001$, $n\geq18$.

C. 1 h uptake/binding of Tf22 (1 μM) in Col is inhibited by unlabeled flg22 (f22, 20 μM). $P<0.001$, $n\geq18$.

D. Overnight accumulation of Tf22 (0.5 μM) in Col is inhibited by unlabeled flg22 (20 μM). $P<0.01$, $n\geq14$.

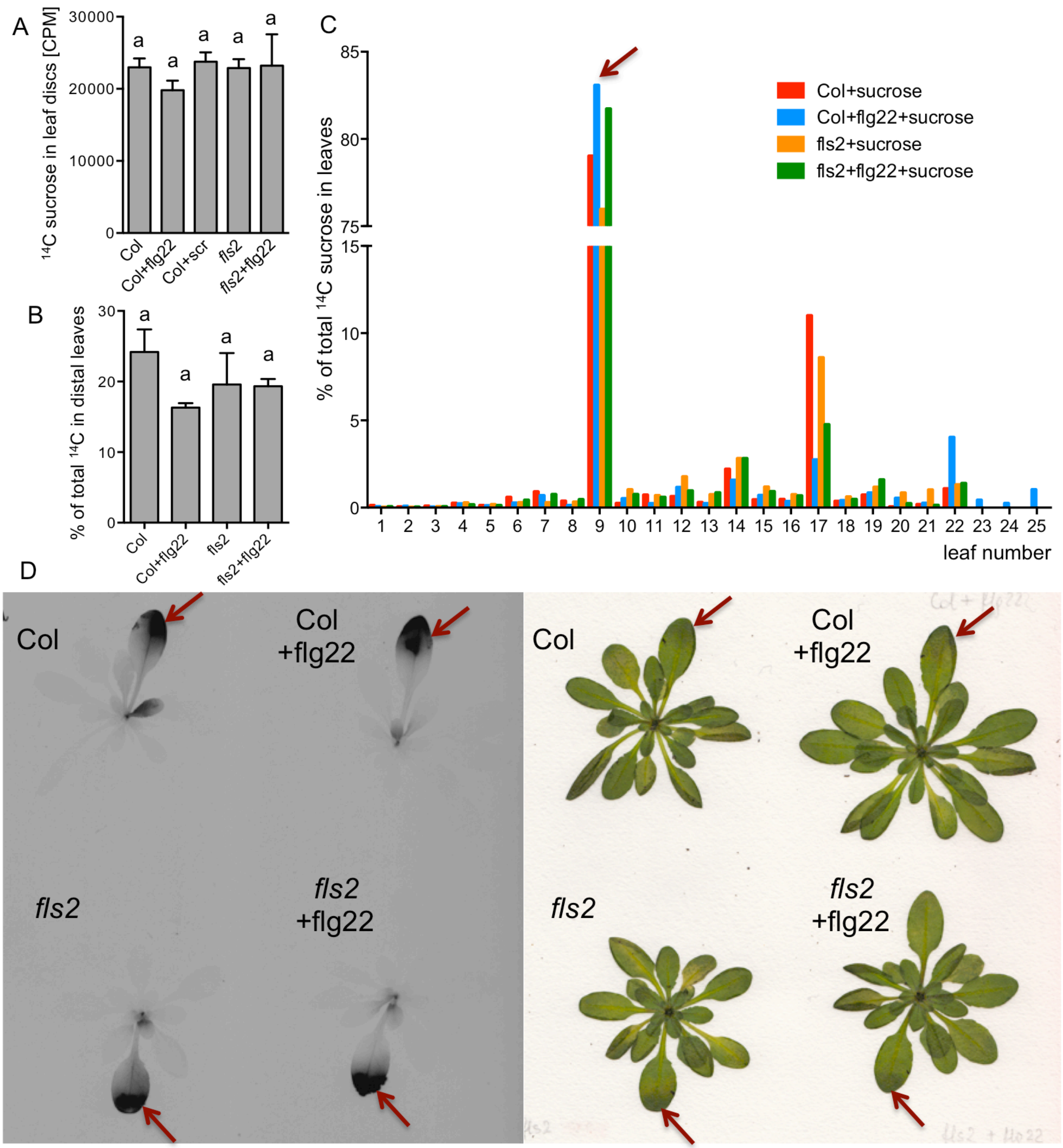

Supplementary Fig. S10. Defense activation does not increase bulk phloem flow.
A. Sucrose uptake is not affected by flg22 or FLS2. Leaf discs were floated on 5 µCi/ml ^{14}C-sucrose with or without 2 µM unlabeled peptide for 1.5 h and washed for 1 h. Mean radioactivity in 9 leaf discs from 2 experiments is shown except Col+scrambled (scr) tested in 1 experiment with 7 leaf discs (P>0.05, ANOVA/Tukey's test).
B-D. Sucrose transport is not affected by flg22 or FLS2. 0.2 µCi of ^{14}C-sucrose +/- 10 µM flg22 was applied on abaxial side of leaf #9 (arrow) of plants grown in soil and imaged by autoradiography after 16h (D). Radioactivity in individual leaves quantified by scintillation counting is shown as percentage of total radioactivity in a plant (C). Percentage of total radioactivity present in distal leaves quantified in 2 experiments is shown in B (P>0.05, ANOVA/Tukey's test).
